# Activation of iNKT Cells Facilitates Liver Repair After Hepatic Ischemia Reperfusion Injury Through Acceleration of Macrophage Polarization

**DOI:** 10.3389/fimmu.2021.754106

**Published:** 2021-10-06

**Authors:** Takuya Goto, Yoshiya Ito, Masashi Satoh, Shuji Nakamoto, Nobuyuki Nishizawa, Kanako Hosono, Takeshi Naitoh, Koji Eshima, Kazuya Iwabuchi, Naoki Hiki, Hideki Amano

**Affiliations:** ^1^ Department of Molecular Pharmacology, Graduate School of Medical Sciences, Kitasato University, Sagamihara, Japan; ^2^ Department of Pharmacology, Kitasato University School of Medicine, Sagamihara, Japan; ^3^ Department of Lower Gastrointestinal Surgery, Kitasato University School of Medicine, Sagamihara, Japan; ^4^ Department of Immunology, Kitasato University School of Medicine, Sagamihara, Japan; ^5^ Department of General Pediatric Hepatobiliary Pancreatic Surgery, Kitasato University School of Medicine, Sagamihara, Japan; ^6^ Department of Upper Gastrointestinal Surgery, Kitasato University School of Medicine, Sagamihara, Japan

**Keywords:** liver, iNKT cells, repair, macrophage, polarization

## Abstract

Macrophage polarization is critical for liver tissue repair following acute liver injury. However, the underlying mechanisms of macrophage phenotype switching are not well defined. Invariant natural killer T (iNKT) cells orchestrate tissue inflammation and tissue repair by regulating cytokine production. Herein, we examined whether iNKT cells played an important role in liver repair after hepatic ischemia-reperfusion (I/R) injury by affecting macrophage polarization. To this end, we subjected male C57BL/6 mice to hepatic I/R injury, and mice received an intraperitoneal (*ip*) injection of α-galactosylceramide (α-GalCer) or vehicle. Compared with that of the vehicle, α-GalCer administration resulted in the promotion of liver repair accompanied by acceleration of macrophage differentiation and by increases in the numbers of Ly6C^high^ pro-inflammatory macrophages and Ly6C^low^ reparative macrophages. iNKT cells activated with α-GalCer produced interleukin (IL)-4 and interferon (IFN)-γ. Treatment with anti-IL-4 antibodies delayed liver repair, which was associated with an increased number of Ly6C^high^ macrophages and a decreased number of Ly6C^low^ macrophages. Treatment with anti-IFN-γ antibodies promoted liver repair, associated with reduced the number of Ly6C^high^ macrophages, but did not change the number of Ly6C^low^ macrophages. Bone marrow-derived macrophages up-regulated the expression of genes related to both a pro-inflammatory and a reparative phenotype when co-cultured with activated iNKT cells. Anti-IL-4 antibodies increased the levels of pro-inflammatory macrophage-related genes and decreased those of reparative macrophage-related genes in cultured macrophages, while anti-IFN-γ antibodies reversed the polarization of macrophages. *Cd1d*-deficient mice showed delayed liver repair and suppressed macrophage switching, compared with that in wild-type mice. These results suggest that the activation of iNKT cells by α-GalCer facilitated liver repair after hepatic I/R injury by both IL-4-and IFN-γ-mediated acceleration of macrophage polarization. Therefore, the activation of iNKT cells may represent a therapeutic tool for liver repair after hepatic I/R injury.

## Introduction

Hepatic ischemia-reperfusion (I/R) injury is a major cause of liver damage during liver resection and transplantation. Growing evidence suggests that inadequate liver repair and regeneration culminates in postoperative liver dysfunction, which is associated with poor patient outcomes (1). Hepatic macrophages play an important role in liver repair after acute liver injury, including hepatic I/R injury (2). Macrophage phenotype shift from a pro-inflammatory to a reparative phenotype in the inflammatory liver is crucial for liver repair following hepatic I/R injury (3–6). However, the mechanisms by which the liver is repaired *via* macrophage differentiation remain to be elucidated.

Invariant natural killer T (iNKT) cells have the unique ability to recognize endogenous and exogenous lipids present on the surface molecule CD1 through an invariant T cell receptor (TCR) (7). iNKT cells can rapidly and robustly produce cytokines, including interferon (IFN)-γ and interleukin (IL)-4, which shape subsequent immune responses upon activation (8). A prototypic ligand for the iNKT cell TCR, α-galactosylceramide (α-GalCer) (9), has been employed extensively to activate iNKT cells, especially as a means to quickly induce large amounts of various cytokines and chemokines (10).

iNKT cells are most frequently found in the liver (11). They constitute 30% of all lymphocytes, patrolling sinusoids constantly in a random fashion (12). Accumulating evidence suggests that the functions of iNKT cells in the pathogenesis of liver disease are complex and that these cells likely play diverse roles. iNKT cells attenuate carbon tetrachloride-induced liver injury associated with neutrophil infiltration (13). During sterile hepatic injury caused by ischemia-reperfusion, there is an influx of iNKT cells, which contributes to tissue damage through IFN-γ production (14). This study demonstrates the activation of liver iNKT cells induced by sterile inflammation, which contributes to tissue injury. In contrast, the pre-activation of iNKT cells by α-GalCer has been reported to protect the liver against I/R injury in mice *via* IL-13 production (15).

iNKT cells mediate acute skin wound healing by producing IFN-γ. Mice lacking iNKT cells show significantly delayed wound closure, with lesser collagen and α-smooth muscle actin deposition and impaired new vessel formation due to the defective induction of IFN-γ from iNKT cells (16). iNKT cells accumulate into the injured regions induced by acute myocardial infarction and cardiac I/R to repair the damaged tissue through IL-10 production (17, 18). Again, in the liver, recent evidence suggests that iNKT cells play a critical role in the promotion of liver tissue repair after focal sterile thermal injury at the surface of the liver through monocyte transition from an inflammatory to a reparative phenotype through IL-4 production (19). On the other hand, iNKT cell activation inhibits liver regeneration after partial hepatectomy in mice, which is mediated through the secretion of both IFN-γ and IL-4 from increased iNKT cells (20).

Accumulating evidence indicates that iNKT cells contribute to the induction of acute sterile inflammation through interaction with macrophages; however, few studies have shown the role of interaction between iNKT cells and monocytes/macrophages in the resolution of inflammation and repair after injury (21). A critical role for iNKT cells in tissue repair is indicated by the highly regulated interactions between iNKT and CD1d expressing cells, which include macrophages and monocytes (22). In addition, macrophage polarization is essential for liver tissue repair after acute liver injury (4). Therefore, the cytokines produced by iNKT cells due to crosstalk with macrophages in the hepatic microenvironment may be involved in macrophage phenotype switching and promote liver repair.

iNKT cells appear to play mitigating roles in various inflammatory disease models through the representative cytokines depending on each model. Here, we attempted to examine the role of iNKT cells in liver repair after hepatic I/R injury in mice by blocking either IFN-γ or IL-4. The facilitation of liver repair after hepatic I/R injury by the activation of iNKT cells with α-GalCer is discussed with an emphasis on the acceleration and enhancement of macrophage phenotype switching.

## Materials and Methods

### Animals

Male C57BL/6 WT mice (8-week-old, 20–25 g) were obtained from Clea Japan (Tokyo, Japan). Male *Cd1d*-deficient (Cd1d^-/-^) mice (8-week-old) were generated as described previously (23, 24). Mice were maintained at constant humidity (50% ± 5%) and temperature (25°C ± 1°C) on a 12 h light/dark cycle, and were provided food and water *ad libitum*. All experimental procedures were approved by the Animal Experimentation and Ethics Committee of the Kitasato University School of Medicine (2020–111) and were performed in accordance with institutional guidelines for animal experimentation, which are based on the Guidelines for Proper Conduct of Animal Experiments published by the Science Council of Japan.

### Animal Procedures

The animals underwent either sham surgery or I/R. Partial hepatic ischemia was induced as previously described (3). Briefly, mice were anesthetized with a combination cocktail containing 0.3 mg/kg of medetomidine hydrochloride (Nippon Zenyaku Kogyo Co., Ltd., Fukushima, Japan), 4.0 mg/kg of midazolam (Astellas Pharma Inc., Tokyo, Japan), and 5.0 mg/kg of butorphanol (Meiji Seika Pharma Co., Ltd., Tokyo, Japan). Laparotomy was performed and the blood supply to the median and left hepatic lobes was occluded for 60 min using an atraumatic vascular clamp (ROBOZ Surgical Instrument, Washington, DC, USA). Reperfusion was initiated by removing the clamps. After reperfusion was initiated, 4 mL/kg of physiological saline was injected and the abdomen was closed with 4-0 nylon sutures. After the procedure, the effect of medetomidine was reversed by *ip* injection of 0.75 mg/kg atipamezole (Nippon Zenyaku Kogyo Co., Ltd.), and the animals were allowed to recover. Sham control mice underwent the same protocol without vascular occlusion.

### Experimental Protocols

Mouse livers were subjected to ischemia followed by reperfusion. Blood was drawn and livers were excised at 6, 24, 48, 72, and 96 h after reperfusion. Serum was used to determine alanine aminotransferase (ALT) activity using a Dri-Chem 7000 Chemistry Analyzer System (Fujifilm, Tokyo, Japan). Immediately after blood collection, the livers were excised and rinsed with saline. A small section of each liver was placed in 10% formaldehyde, and the remaining liver was frozen in liquid nitrogen and stored at -80°C.

Animals received α-GalCer (0.1 mg/kg body weight, *ip*; Funakoshi, Tokyo, Japan) dissolved in 0.1% DMSO and diluted in phosphate-buffered saline (PBS) at the induction of ischemia. The dose of α-GalCer was chosen based on our previous results (25).

In separate experiments, mice were injected *ip* with 500 μg of a neutralizing monoclonal antibody specific for mouse IL-4 (BioLegend, San Diego, CA, USA) (26) or 500 μg of a neutralizing monoclonal antibody specific for mouse IFN-γ (BioLegend) (27) 1 h before the induction of ischemia. As a control group, mice were administered isotype-matched control IgG (BioLegnd).

### Histology and Immunohistochemistry

Excised liver tissues were fixed immediately with buffered 10% formaldehyde for histological analysis. Sections (4 µm thick) were prepared from paraffin-embedded tissues and subjected to either hematoxylin and eosin (H&E) staining or immunostaining. Images of H&E-stained sections were captured using a microscope (Biozero BZ-700 Series; Keyence, Osaka, Japan). The level of necrosis (as a percentage of the total area) was estimated by measuring the necrotic area relative to the entire histological section using the ImageJ software (US National Institutes of Health, Bethesda, MD, USA). The results were expressed as percentages.

Sections were also stained with an antibody against proliferating cell nuclear antigen (PCNA; Thermo Fisher Scientific, Waltham, MA, USA). Immune complexes were detected using Histofine Simple Stain MAX PO (MULTI) (Nichirei, Tokyo, Japan), following the manufacturer’s protocol. The number of PCNA^+^ hepatocytes was counted in six fields (400×) per animal. The percentage of PCNA^+^ hepatocytes was then calculated, and the results were expressed as percentages.

### Immunofluorescence Staining

Tissue samples were fixed with periodate-lysine-paraformaldehyde fixative at room temperature for 3 h. Following cryoprotection with 30% sucrose prepared in 0.1 M phosphate buffer (pH 7.2), fixed liver samples were embedded in Tissue-Tek O.C.T. compound (Sakura Finetek USA, Torrance, CA, USA) and frozen at –80°C. Samples were cut into 8 μm sections using a cryostat and incubated for 1 h at room temperature with Dako Protein Block Serum-Free solution (Glostrup, Denmark) to block any nonspecific binding. The sections were then incubated overnight at 4°C with a rat anti-CD1d monoclonal antibody (BD Bioscience, San Jose, CA, USA) and a rabbit anti-F4/80 polyclonal antibody (Cell Signaling Technology, Beverly, MA, USA). After washing three times in PBS, the sections were incubated for 1 h at room temperature with the following secondary antibodies: Alexa Fluor 488-conjugated donkey anti-rabbit IgG and Alexa Fluor 594-conjugated donkey anti-rat IgG (Invitrogen, Carlsbad, CA, USA). Images of the stained sections were captured using a fluorescence microscope (Biozero BZ-700; Keyence).

### Real-Time Quantitative RT-PCR Analysis

Total RNA was extracted from mouse tissues and homogenized using TRIzol reagent (Thermo Fisher Scientific, Waltham, MA, USA). Single-stranded cDNA was generated from 1 μg of total RNA by reverse transcription using the ReverTra Ace qPCR RT kit (Toyobo, Osaka, Japan), according to the manufacturer’s instructions. Quantitative PCR was performed using TB Green Premix Ex Taq II (Tli RNaseH Plus; Takara Bio, Shiga, Japan). The gene-specific primers used for real-time RT-PCR were designed using the Primer 3 software (http://primer3.sourceforge.net/) based on data from GenBank. The primer sequences are listed in **Table 1**. Data were normalized to the expression of glyceraldehyde-3-phosphate dehydrogenase (GAPDH) in each sample.

**Table 1:** Primers used for reverse transcription and quantitative PCR.

Gene	Forward primer sequence (5’-3’)	Reverse primer sequence (5’-3’)
*Tnf*	TCTTCTCATTCCTGCTTGTGG	GATCTGAGTGTGAGGGTCTGG
*Il1b*	TACATCAGCACCTCACAAGCA	CCAGCCCATACTTTAGGAAGA
*Il6*	CAAAGCCAGAGTCCTTCAGAG	TAGGAGAGCATTGGAAATTGG
*Nos2*	AAAACCCCTTGTGCTGTTCTC	CTGGAACATTCTGTGCTGTCC
*Ifng*	ATCTGGAGGAACTGGCAAAAG	CGGAACCAAATGAGATCAGAA
*Mrc1*	TTTGTCCATTGCACTTTGAGG	TGCCAGGTTAAAGCAGACTTG
*Retnla*	TGCCAATCCAGCTAACTATCC	CACACCCAGTAGCAGTCATCC
*Il4*	GAACGAGGTCACAGGAGAAGG	CTTGGAAGCCCTACAGACGAG
*Il13*	CAGCATGGTATGGAGTGTGG	TGGGCTACTTCGATTTTGGT
*Cd1d1*	ACTCAGCCACCATCAGCTTC	AGGGTACATTTCACAGCCCG
*Itgam*	ACTGGAGCAAGAATAGGAAGG	ATAGTCTGGGTTGGGAACAGG
*Cd3e*	TCCTGCGCCTCAATTATACAC	CTGAGCATCCATAGCCAGAAC
*Gapdh*	ACATCAAGAAGGTGGTGAAGC	AAGGTGGAAGAGTGGGAGTTG

### Isolation of Intrahepatic Leukocytes

Under anesthesia induced by *ip* injection of a mixture of 0.75 mg/kg medetomidine, 4.0 mg/kg midazolam, and 5.0 mg/kg butorphanol, the liver was perfused with 10 mL of 1× Hank’s balanced salt solution *via* the portal vein. The excised livers were immediately placed in RPMI at room temperature, minced using scissors, and incubated at 37°C for 20 min in RPMI containing 0.05% collagenase (Type IV; Sigma Chemical Co., St. Louis, MO, USA). The tissues were then pressed through a 70 μm cell strainer. Cells were centrifuged at 1290 × g for 10 min at 4°C. Pelleted cells were resuspended in PBS. Non-parenchymal cells were isolated from liver homogenates by density-gradient centrifugation on 33% Percoll (GE Healthcare Life Sciences, Piscataway, NJ, USA), as previously reported (28). Viable nucleated cells were counted by trypan blue exclusion and resuspended at a uniform cell density.

### Flow Cytometric Analysis and Cell Sorting

The cells were incubated with the 2.4G2 mAb (anti-cγRIII/II) to block non-specific binding of the primary mAb. Then, the cells were stained with a combination of the following fluorochrome-conjugated antibodies: FITC-conjugated anti-TCRβ (H57-597, BioLegend), anti-F4/80 (BM8, BioLegend), APC-conjugated anti-CD1d antibody (1B1, BioLegend), APC/CY7-conjugated anti-Ly6G (1A8, BioLegend), PE/Cy7-conjugated anti-CD11b (M1/70, BioLegend), PE-conjugated anti-CD45 (30-F11, BioLegend), Brilliant Violet 510-conjugated anti-Ly6C (HK1.4, BioLegend), Brilliant Violet 421-conjugated anti-CD45 (30-F11, BioLegend), and α-GalCer (PBS-57)-loaded CD1d tetramer and control CD1d-tetramer were kindly provided by The NIH Tetramer Core Facility (NTCF) at Emory University (Atlanta, GE, USA). Tubes containing the mixture were placed in the dark on ice for 30 min. Pellets were washed twice with PBS. For flow cytometric analysis, cells were initially gated on forward scatter (FSC) and side scatter (SSC), and then gated on CD45^+^ cells. Cells positive for 7-aminoactinomycin D (BioLegend), which stains dead cells, were gated electronically and removed from the analysis. Samples were analyzed on a FACSVerse cytometer (BD Biosciences, Franklin Lakes, NJ, USA). Data were analyzed using Kaluza software v2.1 (Beckman Coulter, Brea, CA, USA). We quantified and the cells as a percentage of total live CD45^+^ cells (CD45^+^7ADD^-^ cells).

For intracellular cytokine staining, cells suspensions from liver tissue were stained for surface marker expression before fixation and permeabilization using the FOXP3 Fix/Perm Buffer Set (BioLegend). Treated cells were stained with APC-conjugated anti-IL-4 mAb (11B11, BioLegend), anti-IFN-γ mAb (XMG1.2, BioLegend), and PE/Cy7 conjugated anti-IL-13 mAb (eBio 13A, eBioscience, San Diego, CA, USA). Stained cells were acquired with FACS Verse flow cytometers (BD Biosciences) and analyzed using the FlowJo software (FlowJo, LLC, Ashland, OR, USA).

### Cell Culture

BM cells were isolated from the femurs and tibias of 8-week-old mice. The bones were flushed with PBS and the erythrocytes were lysed by treatment with RBC lysis buffer (BioLegend). BM cells were cultured in RPMI 1640 medium containing 10% fetal bovine serum and plated in 6-well plates at a density of 1 × 10^6^/mL. Cultures were incubated at 37°C in a humidified atmosphere containing 5% CO_2_. To generate BM-derived macrophages, cells were cultured for 7 days in the presence of 20 ng/mL murine recombinant macrophage colony-stimulating factor (BioLegend). iNKT cells (TCRβ^+^α-GalCer-loaded CD1d tetramer^+^ cells) were purified by cell sorting using a BD FACSAria™ II (BD Biosciences). For the co-culture experiments, sorted hepatic iNKT cells (1 × 10^5^/well) and macrophages on day 7 of culture (3 × 10^5^/well) were seeded into 12-well plates in RPMI 1640 medium supplemented with 5% FCS. The co-culture was maintained in the presence of α-GalCer (100 ng/mL) for 72 h (29). Antibody-mediated neutralization experiments were performed in the presence of an additional 5 µg/mL of anti-mouse IL-4 monoclonal antibody (BioLegend) (30), 5 µg/mL of anti-mouse IFNγ monoclonal antibody (BioLegend) (31), or 5 µg/mL of isotype control IgG (BioLegend).

Both non-adherent (floating cells) and adherent cells were then harvested. The cells were homogenized in TRIzol (Life Technologies) and their mRNA levels were measured using real-time RT-PCR. Supernatants from co-cultured iNKT cells and macrophages were collected to measure cytokine levels using the cytometric bead array (BD Bioscience) and data were analyzed using the BD Biosciences FCAP software (V3.0).

### Statistical Analysis

All results are presented as mean ± standard deviation (SD). All statistical analyses were performed using the GraphPad Prism software, version 8 (GraphPad Software, La Jolla, CA, USA). Data were compared between two groups using unpaired two-tailed Student’s t-tests and between multiple groups using one-way analyses of variance followed by Tukey’s *post-hoc* tests. A p-value < 0.05 was considered statistically significant. Significance is depicted with asterisks on graphs as follows: *P < 0.05, **P < 0.01, ***P < 0.001, and ****P < 0.0001.

## Results

### Activation of iNKT Cells Facilitated Liver Repair after Hepatic I/R Injury

**Figure 1:**
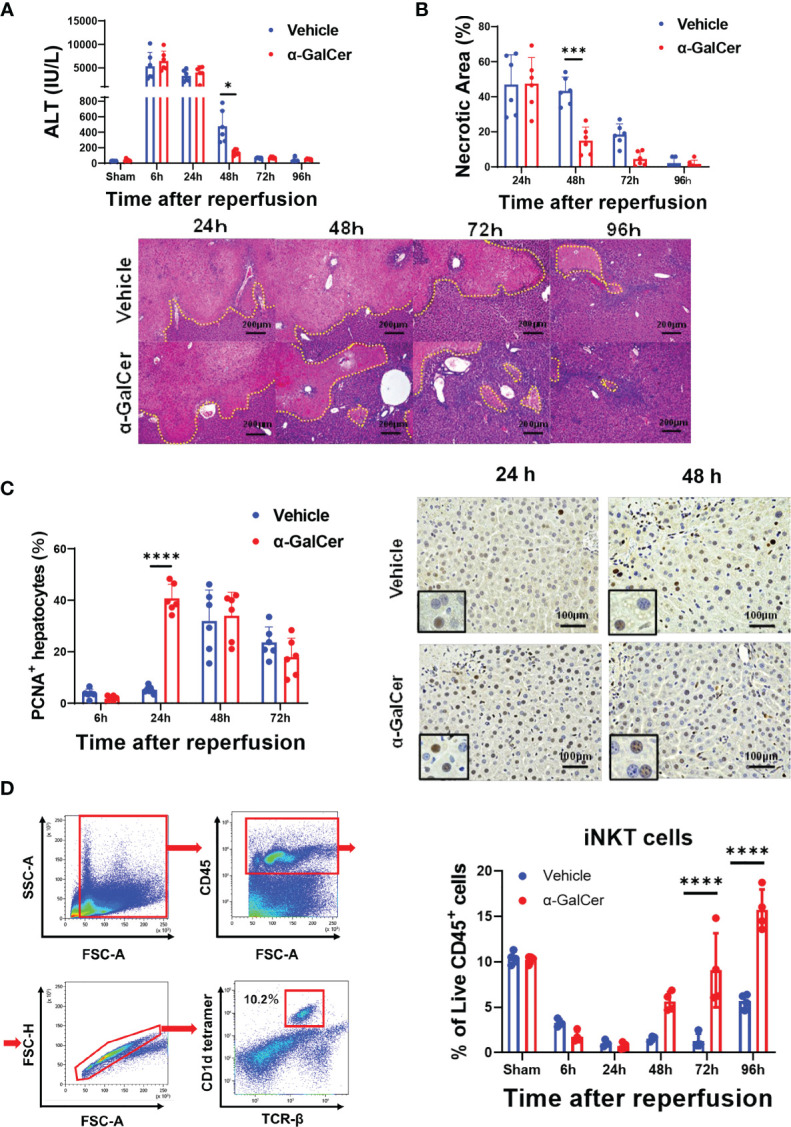
Activation of iNKT cells facilitates liver repair after hepatic I/R injury. **(A, B)** Changes in ALT levels **(A)** and hepatic necrotic area **(B)** in mice treated with α-GalCer and vehicle after hepatic I/R injury. Data are expressed as the mean ± SD (six biological replicates per group). *P < 0.05, ***P < 0.001. Representative photomicrographs of H&E-stained liver sections from mice treated with α-GalCer or vehicle after hepatic I/R injury. Scale bars, 200 μm. **(C)** Changes in PCNA^+^-hepatocytes in mice treated with α-GalCer or vehicle. Data are expressed as the mean ± SD (six biological replicates per group). ****P < 0.0001. Representative images showing immunohistochemical staining of PCNA in mice treated with α-GalCer or vehicle at 24 h and 48 h post-reperfusion. Large and round cells are considered hepatocytes, and typical labeled hepatocytes are shown at the lower left corner. Scale bar, 100 μm. **(D)** Representative dot plots and the gating strategy for iNKT cells in sham-operated mice. Changes in hepatic iNKT cells in mice treated with α-GalCer or vehicle after hepatic I/R injury. Data are expressed as the mean ± SD (four biological replicates per group). ****P < 0.0001.

To examine whether activation of iNKT cells facilitated liver repair after hepatic I/R injury, mice were treated with α-GalCer at the induction of ischemia of the liver. In mice treated with α-GalCer or the vehicle, ALT levels peaked at 6 h post-reperfusion and decreased gradually (**Figure 1A**). There was no statistically significant difference in ALT levels at 6 h and 24 h between the two groups. At 48 h post-reperfusion, ALT levels in α-GalCer-treated mice were lower than those in vehicle-treated mice. Consistently, changes in hepatic necrosis area were similar to those in ALT levels at 24 h (**Figure 1B**), while the necrotic area in α-GalCer-treated mice at 48 h post-reperfusion was lower than that in vehicle-treated mice (**Figure 1B**). We also employed immunocytochemistry for PCNA to examine hepatocyte proliferation during hepatic I/R injury. In vehicle-treated mice, the percentage of PCNA^+^-hepatocytes increased 48 h post-reperfusion, which is consistent with the results in previous reports showing that the liver enters a proliferative phase 48 h post-reperfusion (3). In contrast, the number of PCNA^+^-hepatocytes in α-GalCer-treated mice began to increase at 24 h post-perfusion, and the percentage of PCNA^+^ hepatocytes in α-GalCer-treated mice was higher than that in vehicle-treated mice at 24 h (**Figure 1C**). These results indicated that α-GalCer administration appeared to accelerate the resolution of live inflammation and liver repair after hepatic I/R injury, as evidenced by decreases in ALT levels and necrotic area, and increases in the number of PCNA^+^-hepatocytes.

In sham-operated mice treated with α-GalCer, ALT levels were moderately elevated at 24 h and 48 h after sham-operation and decreased at 72 h (**Figure S1**). The ALT levels in sham-operated mice were remarkably lower than those in α-GalCer-treated mice subjected to hepatic I/R injury. Although H&E staining of the livers obtained from sham-operated mice treated with α-GalCer exhibited the formation of small inflammatory foci, hepatic necrosis similar to that observed in mice subjected to hepatic I/R injury (**Figure 1B**) was not observed in sham-operated mice. In addition, PCNA expression in the hepatocytes was not up-regulated.

When iNKT cells were identified as TCRβ^+^ and CD1d-tetramer^+^, using flow cytometry analysis (**Figure 1D**, left panel), the percentage of iNKT cells in total live CD45^+^ cells was decreased at 6 h post-reperfusion and markedly decreased at 24 h and 48 h post-reperfusion in both α-GalCer- and vehicle-treated mice (**Figure 1D**, right panel). Although the population of iNKT cells in vehicle-treated mice remained low at 72 h post-reperfusion, it was slightly elevated at 96 h post-reperfusion. In contrast, the percentage of iNKT cells in α-GalCer-treated mice at 72 and 96 h was larger than that in vehicle-treated mice.

### Genes Expression of Pro-Inflammatory and Anti-Inflammatory Mediators

**Figure 2:**
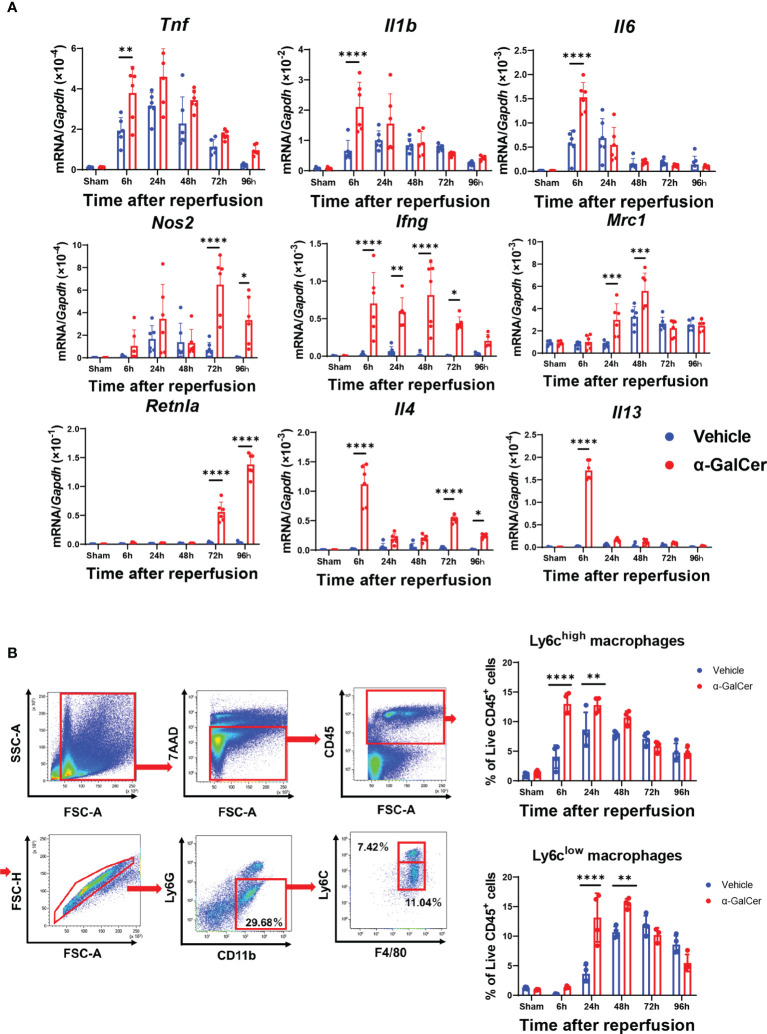
Changes in expression of genes related to pro-inflammatory and reparative macrophage phenotypes and in infiltration of macrophages during hepatic I/R injury. **(A)** Expression of mRNA encoding genes related to a pro-inflammatory macrophage phenotype (*Tnf*, *Il1b, Il6, Nos2*, and *Ifng*) and genes related to a reparative macrophage phenotype (*Mrc1, Retnla, Il4*, and *Il13*) in mice treated with α-GalCer or vehicle. Data are expressed as the mean ± SD (six biological replicates per group). *P < 0.05, **P < 0.01, ***P < 0.001, ****P < 0.0001. **(B)** Flow cytometry gating strategy used to identify liver macrophages in mice treated with α-GalCer at 48 h post-reperfusion. After gating out Ly6G^+^ and CD11b^+^ cells, cells were separated into these two subsets based on expression of Ly6C and F4/80. Ly6G^-^/Ly6C^high^/CD11b^high^/F4/80^high^ cells are defined as Ly6C^high^ macrophages (pro-inflammatory macrophages) and Ly6G^-^/Ly6C^low^/CD11b^high^/F4/80^high^ cells are defined as Ly6C^low^ macrophages (reparative macrophages). Changes in the percentage of Ly6C^high^ macrophages (Ly6G^-^/Ly6C^high^/CD11b^high^/F4/80^high^ cells) and Ly6C^low^ macrophages (Ly6G^-^/Ly6C^low^/CD11b^high^/F4/80^high^) in livers from mice treated with α-GalCer or vehicle after hepatic I/R injury. Data are expressed as the mean ± SD (four biological replicates per group). **P < 0.01, ****P < 0.0001.

During the injury and repair phases of hepatic I/R injury, genes related to pro-inflammatory macrophages and reparative macrophages in liver tissues are expressed (3, 28). Accordingly, we compared the expression of mRNA encoding pro-inflammatory macrophage-related genes (*tumor necrosis factor*, *Tnf; interleukin-1β*, *Il1b*; *interleukin-6*, *Il6*; nitric oxide synthetase-2, *Nos2*; and interferon-γ, *Ifng*) and mRNA encoding reparative macrophage-related genes (mannose receptor, *Mrc1*; *resistin-like molecule*-α, *Retnla*; interleukin-4, *Il4*; and *interleukin-13*, *Il13*) between the two groups (**Figure 2A**). The mRNA levels of pro-inflammatory macrophages, including *Tnf, Il1b, Il6, and Ifng*, in the livers of α-GalCer-treated mice at 6 h post-reperfusion were higher than those in the livers of vehicle-treated mice. In addition, the mRNA levels of *Nos2* and *Ifng* in α-GalCer-treated mice increased during the repair phase of hepatic I/R injury. With respect to the genes related to reparative macrophages, the levels of *Mrc1, Retnla*, and *Il4* in α-GalCer-treated mice in the repair phase of hepatic I/R injury were higher than those in vehicle-treated mice. Further, the expression levels of mRNAs encoding IL-4 and IL-13 in the livers of α-GalCer-treated mice at 6 h post-reperfusion were higher than those in the livers of vehicle-treated mice.

### Accumulation of Macrophages During Hepatic I/R Injury

To further identify macrophage subsets during hepatic I/R injury, Ly6G^high^ and CD11b^+^ populations were gated out from live CD45^+^ cells to exclude neutrophils. Next, we examined the subpopulations of liver macrophages isolated from mice treated with α-GalCer or the vehicle (**Figure 2B**) and found two different phenotypes of macrophages: pro-inflammatory macrophages, namely the Ly6G-/Ly6C^high^/CD11b^high^/F4/80^high^ cells (Ly6C^high^ macrophages), and reparative macrophages, namely the Ly6G-/Ly6C^low^/CD11b^high^/F4/80^high^ cells (Ly6C^low^ macrophages). Flow cytometry analysis revealed that the percentage of Ly6C^high^ macrophages in vehicle-treated livers was elevated at 6 h, and these levels were sustained until they decreased at 96 h (**Figure 2B**). The percentage of Ly6C^high^ macrophages at 6 h and 24 h post-reperfusion in α-GalCer-treated livers was further increased than that in vehicle-treated livers (**Figure 2B**). The time course of changes in Ly6C^high^ macrophages in α-GalCer-treated livers appeared to be similar to that in vehicle-treated livers (**Figure 2B**).

Regarding the changes in the reparative macrophages, the percentage of Ly6C^low^ macrophages in vehicle-treated livers appeared to increase from 24 h, reaching maximal levels at 72 h (**Figure 2B**). In contrast, the percentage of Ly6C^low^ macrophages in the livers of α-GalCer-treated mice showed significant increase at 24 h, reached a peak at 48 h, and then decreased. Taken together, these results indicate that α-GalCer treatment increased both pro-inflammatory and reparative macrophage populations and induced early switching from a pro-inflammatory to a reparative macrophage phenotype during hepatic I/R injury.

Flow cytometry also revealed neutrophils (Ly6G^+^/CD11b^+^) to be the major infiltrating leukocyte population in livers exposed to hepatic I/R injury (**Figure S2B**). The accumulation of neutrophils was evident and similar at 6 h post-reperfusion in both α-GalCer- and vehicle-treated livers. The neutrophil level subsequently decreased, reaching a nadir at 96 h post-reperfusion. At 48 h and 72 h post-reperfusion, the percentage of neutrophils in α-GalCer-treated mice was lower than that in vehicle-treated mice. KC populations (Ly6G-/Ly6C-/CD11b^high^/F4/80^high^) in mice treated with either α-GalCer or the vehicle were markedly reduced after hepatic I/R injury. KCs in vehicle-treated mice began to re-appear and gradually increased in population at 72 h and 96 h post-reperfusion (**Figure S2C**). The populations of KCs in α-GalCer-treated mice remained low after hepatic I/R injury, and the levels at 96 h were lower than those in vehicle-treated mice.

### iNKT Cells Produced IL-4 and IFN-γ in the Early Phase of Hepatic I/R Injury

**Figure 3:**
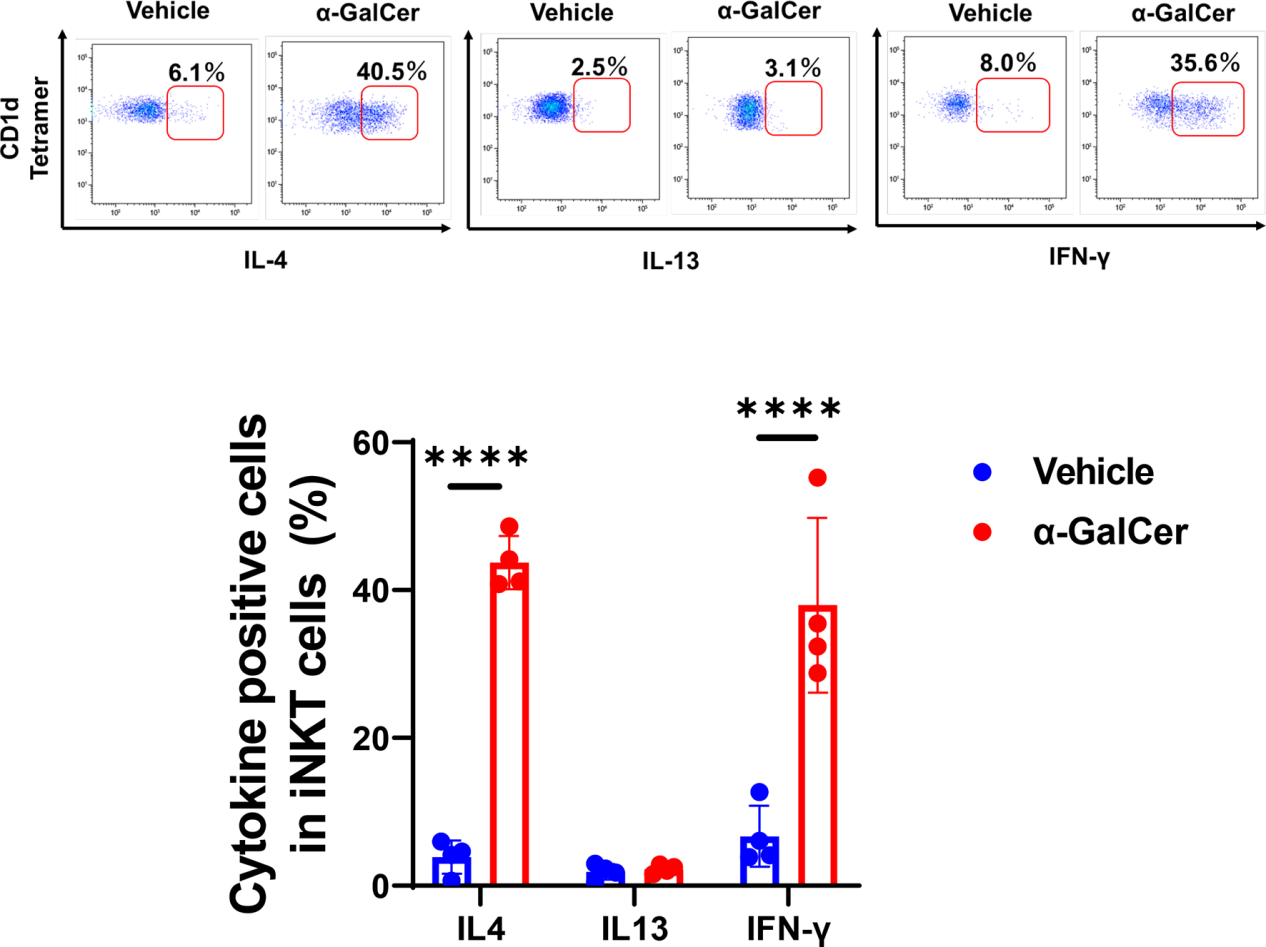
Production of cytokines from iNKT cells after hepatic I/R injury. Representative dot plots in liver iNKT cells at 6 h post-reperfusion (upper panels). The percentage of IL-4-, IL-13-, and IFN-γ-positive cells in iNKT cells in mice treated with α-GalCer or vehicle (lower panel). Data are expressed as the mean ± SD (four biological replicates per group). ****P < 0.0001.

The switch in macrophage phenotypes in the injured region is responsible for liver repair after acute liver injury (2, 32). Cytokines, including IL-4, IL-13, and IFN-γ, are involved in the transition from pro-inflammatory to reparative macrophages. To determine whether iNKT cells played a role in modulating the local reprogramming of macrophages after injury, we measured the levels of these cytokines produced by activated iNKT cells during hepatic I/R injury (**Figure 3**). α-GalCer treatment enhanced iNKT cell-derived IL-4 and IFN-γ levels, but not IL-13 levels, at 6 h post-reperfusion, whereas iNKT cells produced lower levels of these cytokines without of α-GalCer stimulation.

### Blockade of IL-4 Delayed Macrophage Differentiation and Liver Repair

**Figure 4:**
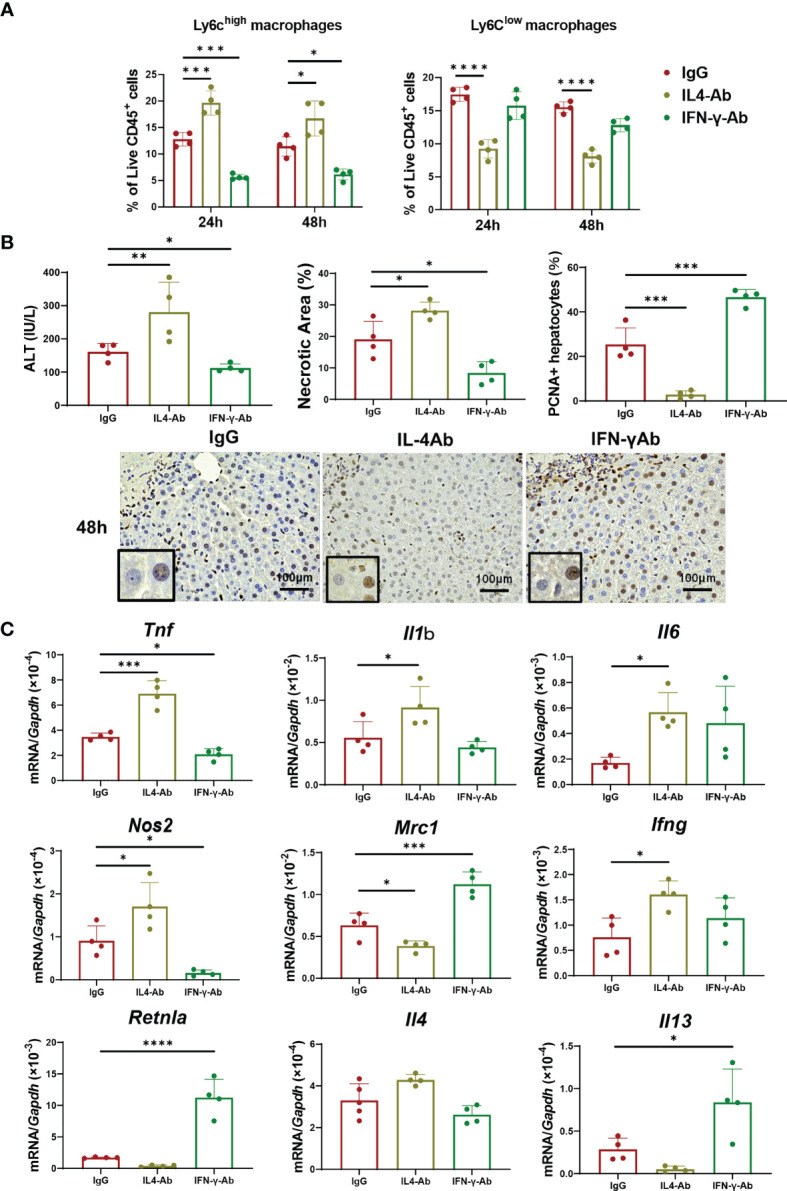
Blockade of IL-4 or IFN-γ suppresses macrophage polarization and liver repair after hepatic I/R injury. Anti-IL-4 or IFN-γ neutralizing antibodies were administered to mice 1 h prior to the induction of ischemia, and α-GalCer was administered at the induction of ischemia. **(A)** The percentage of Ly6C^high^ macrophages (Ly6G^-^/Ly6C^high^/CD11b^high^/F4/80^high^ cells) and Ly6C^low^ macrophages (Ly6G^-^/Ly6C^low^/CD11b^high^/F4/80^high^ cells) in mice treated with anti-IL-4 antibodies, anti-IFN-γ antibodies, or IgG. Data are expressed as the mean ± SD (four biological replicates per group). *P < 0.05, ***P < 0.001, and ****P < 0.0001. **(B)** ALT levels, hepatic necrosis, and PCNA^+^hepatocytes (%) in mice treated with anti-IL-4 antibodies, anti-IFN-γ antibodies, or IgG. Data are expressed as the mean ± SD (four biological replicates per group). *P < 0.05, **P < 0.01, ***P < 0.001. Representative images showing immunohistochemical staining of PCNA in mice treated with anti-IL-4 antibodies, anti-IFN-γ antibodies, or IgG at 48 h post-reperfusion. Large and rounded cells are considered hepatocytes, and typically labeled hepatocytes are shown at the lower-left corner. Scale bar, 100 μm. **(C)** Expression of genes related to pro-inflammatory (*Tnf, Il1b, Il6, Nos2, and Ifng*) and reparative macrophage phenotypes (*Mrc1, Retnla, Il4*, and *Il13*) after hepatic I/R injury in mice treated with anti-IL-4, anti-IFN-γ, or IgG antibodies. Data are expressed as the mean ± SD (four biological replicates per group). *P < 0.05, ***P < 0.001, ****P < 0.0001.

Because IL-4 and/or IFN-γ appear to be involved in macrophage polarization toward a reparative or pro-inflammatory phenotype, respectively, we examined whether these cytokines contributed to stimulating the phenotypic switch of macrophages associated with liver repair (**Figure 4**). First, we examined the effect of IL-4 blockade on macrophage shift and liver repair. Anti-IL-4 neutralizing antibodies or control IgG were administered to mice 1 h before the induction of ischemia concomitant with administration of α-GalCer. Treatment with anti-IL-4 antibodies increased the percentage of Ly6C^high^ macrophages at 24 h and 48 h post-reperfusion and, at the same time, decreased the percentage of Ly6C^low^ macrophages (**Figure 4A**). Additionally, the accumulation of neutrophils in the livers did not differ between the two treatment groups (**Figure S3**). There was also no statistical difference in the KC population between the two treatment groups (**Figure S3B**). Treatment with anti-IL-4 antibodies increased ALT levels and necrotic area and decreased the number of PCNA^+^-hepatocytes at 48 h post-reperfusion (**Figure 4B**). Further, the levels of genes related to pro-inflammatory macrophages, including *Tnf, Il1b, Il6, Nos2*, and *Ifng*, in mice treated with anti-IL-4 antibodies were higher than those in mice treated with control IgG. There were significant differences in the expression of genes related to reparative macrophages, including *Mrc1, Retnla*, and *Il13*, between the two treatments. Collectively, these results suggested that IL-4-mediated stimulation of macrophage polarization from a pro-inflammatory to a reparative phenotype contributes to liver repair.

### Blockade of IFN-γ Decreased Pro-Inflammatory Macrophages and Liver Inflammation

We also wanted to know whether IFN-γ mediates macrophage polarization toward a pro-inflammatory phenotype in α-GalCer-treated mice. To this end, α-GalCer-treated mice were pre-treated with anti-IFN-γ neutralizing antibodies prior to the induction of hepatic I/R injury. Flow cytometry analyses revealed that treatment with anti-IFN-γ antibodies decreased the percentage of Ly6C^high^ macrophages in α-GalCer-treated livers at 24 h and 48 h post-reperfusion compared with that in IgG-treated livers, but no changes in Ly6C^low^ macrophage percentages were observed (**Figure 4A**). These results suggest that IFN-γ might have mediated the switch of macrophages into a pro-inflammatory phenotype, but it did not affect the transition of macrophages into a reparative phenotype. Anti-IFN-γ antibody treatment reduced the percentage of neutrophils at 24 h post-reperfusion, compared with control IgG treatment, but did not change the percentage of KC populations (**Figure S3**).

Treatment with anti-IFN-γ antibodies decreased the ALT levels and necrotic area and increased the number of PCNA^+^-hepatocytes at 48 h post-reperfusion (**Figure 4B**). The expression levels of genes related to pro-inflammatory macrophages, including *Tnf* and *Nos2*, in IFN-γ antibody-treated mice were lower than those in IgG-treated mice. In addition, the expression levels of genes related to reparative macrophages, including *Mrc1*, *Retnla, and Il13*, were higher in IFN-γ antibody-treated mice than in IgG-treated mice.

Taken together, these results suggested that IFN-γ contribute to an increase in the percentage of pro-inflammatory macrophages, which was associated with the enhancement of liver inflammation and suppression of hepatocyte proliferation.

### Expression of CD1d in Macrophages During Hepatic I/R Injury

We examined whether the α-GalCer-activated iNKT cells interacted with macrophages expressing CD1d *in situ*. Immunofluorescence analysis revealed that CD1d was expressed in F4/80^+^ cells (macrophages) at 6 h post-reperfusion (**Figure S4A**). Further, flow cytometry analysis showed that the Ly6C^high^ macrophage populations were strongly positive for CD1d (**Figure S4B**). Because there were few Ly6C^low^ macrophages at 6 h, CD1d expression levels were not detected in these cells. At 24 and 48 h post-reperfusion, both Ly6C^high^ and Ly6C^low^ macrophages expressed CD1d. These results suggested that macrophages were a source of CD1d expression, which interacted with iNKT cells in the early phase of hepatic I/R injury.

### Interaction of Cultured iNKT Cells With Macrophages

Next, we investigated whether activated iNKT cells affected macrophage differentiation using co-culture systems of BM-derived macrophages with iNKT cells (**Figure S5**). First, we determined the characteristics of adherent and non-adherent cells in the culture wells. Adherent cells displayed an increase and decrease in *Itgam* and *Cd3e* levels, respectively, whereas non-adherent cells showed the opposite phenotype (**Figure S5A, B**). These results indicated that adherent cells were recognized as macrophages and that non-adherent cells were iNKT cells in this co-culture system.

**Figure 5:**
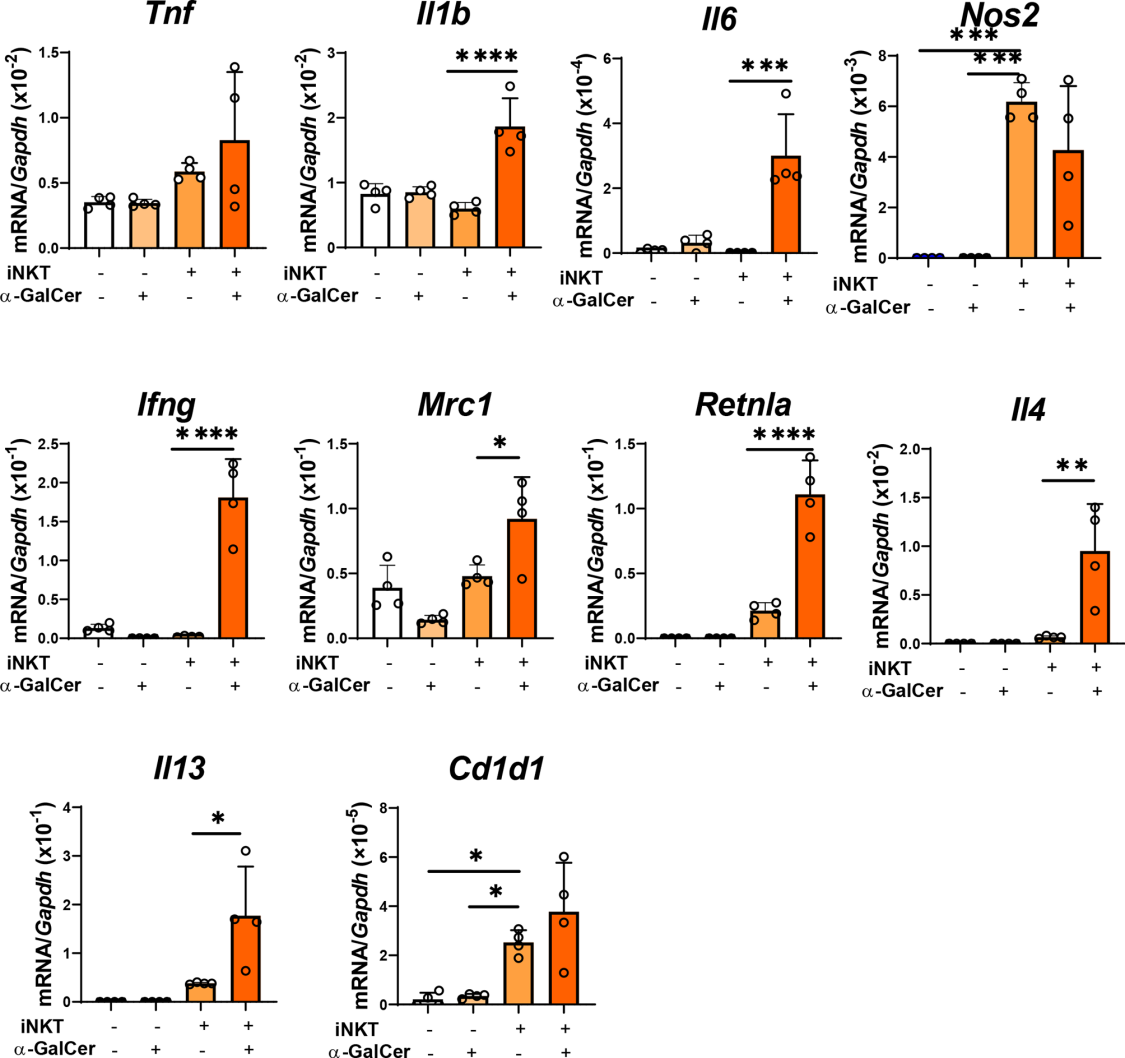
Activated iNKT cells made cultured macrophages differentiate in both ways. The levels of mRNA encoding genes related to a pro-inflammatory macrophage phenotype (*Tnf, Il1b, Il6, Nos2, and Ifng*), genes related to a reparative macrophage phenotype (*Mrc1, Retnla, Il4, and Il13*), and *Cd1d* in macrophages co-cultured with iNKT cells. Macrophages were cultured alone, in the presence of α-GalCer, or with isolated hepatic iNKT cells, or isolated hepatic iNKT cells and α-GalCer for 72 h. Data are expressed as the mean ± SD (four mice per group, representative data from two independent replicates). *P < 0.05, **P < 0.01, ***P < 0.001, ****P < 0.0001.

We also examined whether iNKT cells stimulated a shift in macrophage phenotype *in vitro* (**Figure 5**). Naïve macrophages treated with α-GalCer did not up-regulate the mRNA expressions related to the phenotypes of pro-inflammatory and reparative macrophages. In addition, naïve macrophages co-cultured with iNKT cells in the absence of α-GalCer did not up-regulate the expression of mRNA related to a pro-inflammatory macrophageo phenotype (*Tnf, Il1b, Il6*, and *Ifng, but not Nos2*) or reparative macrophage phenotype (*Mrc1*, *Retnla, Il4,and Il13*). In contrast, naïve macrophages co-cultured with iNKT cells in the presence of α-GalCer increased the expression levels of genes related to a pro-inflammatory macrophage phenotype (*Il1b, Il6*, and *Ifng*) and a reparative macrophage phenotype (*Mrc1*, *Retnla, Il4,and Il13*). These results indicated that activated iNKT cells interacted with macrophages to differentiate them into pro-inflammatory and reparative phenotypes. Further, we examined the expression of CD1d in macrophages. *Cd1d1* was expressed in naïve macrophages, and α-GalCer treatment alone did not affect this expression. The expression of *Cd1d1* in macrophages was up-regulated in macrophages co-cultured with activated iNKT cells, suggesting that the interaction of iNKT cells with macrophages through CD1d converted the macrophage phenotypes.

### Effect of IL-4 and IFN-γ Inhibition on Differentiation of Macrophages Co-Cultured With iNKT Cells

We assessed whether the iNKT cells produced IL-4 and IFN-γ, which polarize the macrophage phenotypes (**Figure S6**). iNKT cells significantly increased the mRNA (**Figure S6A**) and protein levels (**Figure S6B**) of these cytokines in the co-culture system.

**Figure 6:**
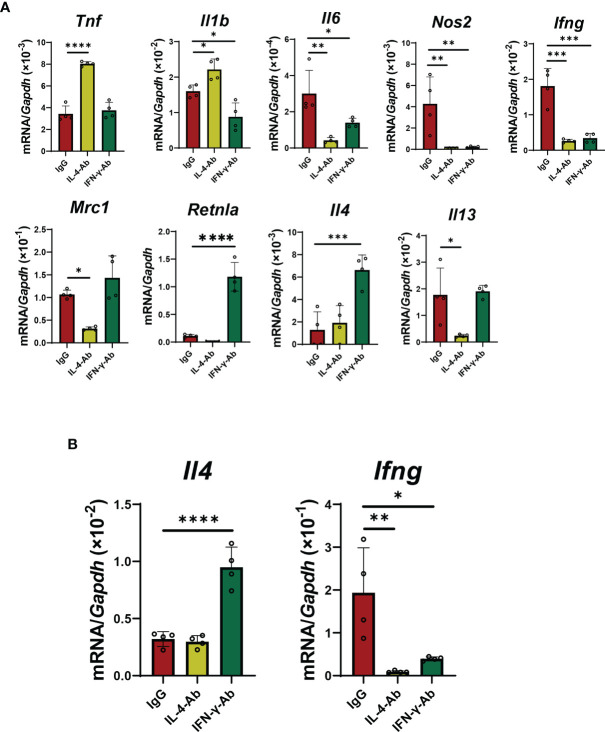
Effects of blockade of IL-4 or IFN-γ on macrophage differentiation and cytokines production from iNKT cells. **(A)** The levels of mRNA encoding genes related to a pro-inflammatory macrophage phenotype (*Tnf, Il1b, Il6, Nos2, and Ifng*) and genes related to a reparative macrophage phenotype (*Mrc1, Retnla, Il4, and Il13*) in macrophages co-cultured with iNKT cells. Macrophages co-cultured with iNKT cells in the presence of α-GalCer were incubated for 72 h with an IL-4- (5 μg/mL) or IL-13-neutralizing antibody (5 μg/mL). Data are expressed as the mean ± SD (four mice per group, representative data from two independent experiments). *P < 0.05, **P < 0.01, ***P < 0.001, ****P < 0.0001. **(B)** The levels of mRNA for *Il4* and *Ifng* from iNKT cells. NKT cells co-cultured with macrophages in the presence of α-GalCer were incubated for 72 h with IL-4 (5 μg/mL) or IL-13 neutralizing antibody (5 μg/mL). Data are expressed as the mean ± SD (four mice per group, representative data from two independent experiments). *P < 0.05, **P < 0.01, ****P < 0.0001.

In co-cultured macrophages with activated iNKT cells, treatment with IL-4 antibodies increased the expression of genes related to pro-inflammatory macrophages, including *Tnf* and *Il1b*, and decreased the expression of genes related to reparative macrophages, including *Mrc1* and *Il13* (**Figure 6A**). However, treatment with IL-4 antibodies decreased the mRNA levels of *Il6* and *Nos2*, but caused no change in *Retnla* and *Il4* levels. Anti-IL-4 antibodies did not change *Il4* mRNA levels, but decreased *Ifng* mRNA levels in iNKT cells (**Figure 6B**). In addition, treatment with anti-IFN-γ antibodies decreased the expression of genes related to a pro-inflammatory macrophage phenotype (*Il1b, Il6, Nos2*, and *Ifng*, but not *Tnf*) in macrophages, and increased the expression of genes related to a reparative macrophage phenotype (*Retnla* and *Il4*, not *Mrc1* and *Il13*) in macrophages (**Figure 6A**). Anti-IFN-γ antibodies increased the mRNA levels of *Il4* from iNKT cells, but decreased mRNA levels of *Ifng* (**Figure 6B**).

### Cd1d1^-/-^ Mice Showed Delayed Liver Repair and Macrophage Differentiation During Hepatic I/R Injury

**Figure 7:**
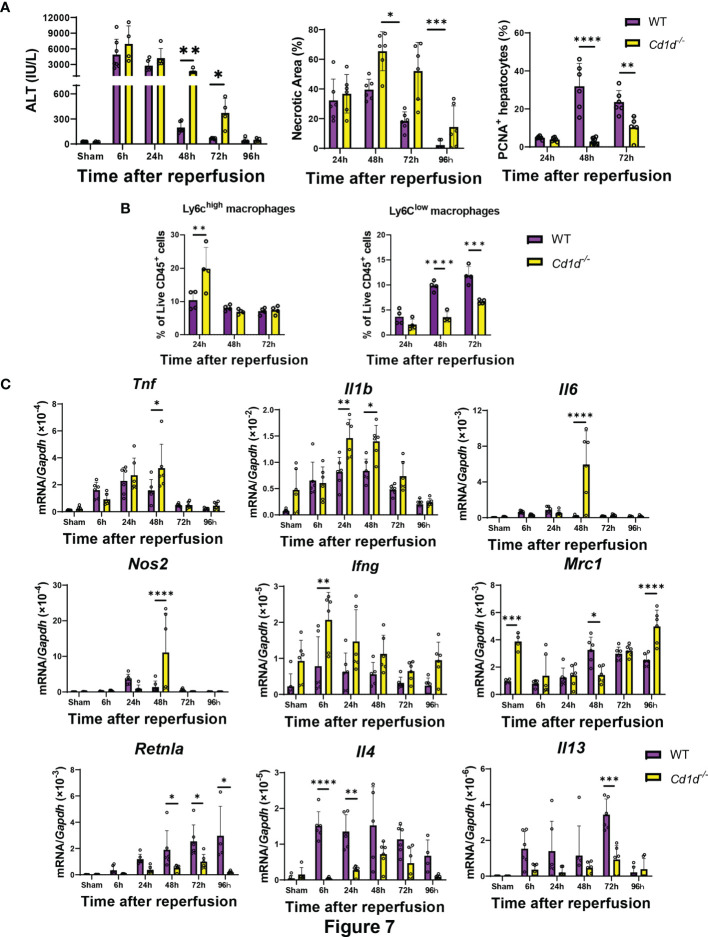
*Cd1d^-/-^* mice exhibited delayed liver repair and macrophage differentiation during hepatic I/R injury. **(A)** ALT levels, hepatic necrosis, and PCNA^+^-hepatocytes (%), **(B)** the percentage of Ly6C^high^ macrophages (Ly6G^-^/Ly6C^high^/CD11b^high^/F4/80^high^ cells) and Ly6C^low^ macrophages (Ly6G^-^/Ly6C^low^/CD11b^high^/F4/80^high^ cells), and **(C)** expression of genes related to pro-inflammatory (*Tnf, Il1b, Il6, Nos2, and Ifng*) and reparative macrophages phenotypes (*Mrc1, Retnla, Il4, and Il13*) after hepatic I/R injury in WT and *Cd1d^-/-^* mice. Data are expressed as the mean ± SD (six biological replicates per group). *P < 0.05, **P < 0.01, ***P < 0.001, ****P < 0.0001.

Finally, we examined whether iNKT-cell deficiency affects liver repair after hepatic I/R injury in *Cd1d^-/-^* mice. *Cd1d^-/-^* mice exhibited delayed liver repair as indicated by increases in ALT levels and hepatic necrotic area and decrease in the number of PCNA^+^-hepatocytes at 48 h and 72 h post-reperfusion (**Figure 7**). Flow cytometry analyses demonstrated that the number Ly6C^high^ macrophages in *Cd1d^-/-^* mice at 24 h was larger than that in WT mice, and that the number of Ly6C^low^ macrophages in *Cd1d^-/-^* mice at 48 h and 72 h was lower than that in WT mice. Expression of genes encoding TNFα, IL-1β, IL-6, and iNOS in the livers of *Cd1d^-/-^* mice was higher than that in the livers of WT mice at 48 h post-reperfusion. In contrast, the expression of mRNAs encoding MR, Fizz1, IL-4, and IL-13 in the livers of *Cd1d^-/-^* mice was lower than that in the livers of WT mice during the repair phase of hepatic I/R injury. In addition, compared with WT mice, *Cd1d^-/-^* mice displayed an increase in mRNA levels of *Ifng* and a decrease in those of *Il4* at 6 h post-reperfusion. To further evaluate the role of iNKT cells in liver repair, *Cd1d^-/-^* mice were treated with α-GalCer or a vehicle as a control. As shown in **Figure S7A**, there were no statistical differences in the levels of ALT, necrotic area, and PCNA^+^-hepatocytes between vehicle- and α-GalCer-treated *Cd1d^-/-^* mice at 72 h post-reperfusion. In addition, the expression levels of genes related to pro-inflammatory and reparative macrophages did not differ between the two treatments (**Figure S7B**).

## Discussion

Accumulation of macrophages into injured regions followed by macrophage polarization is essential for tissue repair after acute liver injury. Late depletion of monocyte-derived macrophages is detrimental to liver regeneration (33). Reparative macrophages facilitate the resolution of necrosis after acute liver injury (34). Although the polarization of macrophages indicates that pro-inflammatory macrophages transition into reparative macrophages at the site of injury, the molecular mechanisms and cell types involved in mediating this phenotypic switch remain unclear (32).

Accumulated evidence indicates that iNKT cells play both protective and injurious roles during acute liver injury. In the present study, we found that the activation of iNKT cells by α-GalCer did not affect the initial hepatic I/R injury, but facilitated liver repair in mice. The liver repair was promoted by the accelerated polarization of macrophages through interaction with iNKT cells. Activated iNKT cells produced both IL-4 and IFN-γ in the early phase of hepatic I/R injury. IL-4 derived from activated iNKT cells induced a switch in macrophage phenotype from pro-inflammatory to reparative to repair the damaged tissues. IFN-γ from activated iNKT cells participated in the rapid accumulation of pro-inflammatory macrophages into the livers after hepatic I/R injury.

α-GalCer is known to activate iNKT cells to rapidly produce various cytokines, such as IL-4, IL-13, and IFN-γ (35). We concluded that α-GalCer-induced activation of iNKT cells based on the significant increase in iNKT-derived cytokine levels, including IL-4 and IFN-γ, observed at 6 h post-reperfusion. Three types of iNKT cells have been identified and including the NKT1, NKT2, and NKT17 cell subsets. NKT1 cells are similar to T helper 1 (TH1) cells which secrete IFN-γ after activation. NKT2 cells secrete cytokines including IL-4 and IL-13 and resemble TH2 cells, whereas NKT17 cells are similar to TH17 cells (11). Therefore, our results indicate that α-GalCer-induced NKT1 and NKT2 were associated with hepatic I/R injury and liver repair. In contrast, iNKT cells in livers exposed to hepatic I/R without α-GalCer stimulation did not produce a large amount of IL-4 and IFN-γ within 6 h post-reperfusion, which is consistent with results from other studies (36). However, Arrenberg et al. (37) showed that 30% of IFN-γ in iNKT cells was present in female mice after 1.5 h hepatic ischemia followed by 6 h reperfusion. The discrepancy may be attributable to the different experimental protocols including the sexual difference in the model used and ischemia duration. Parallelly to these changes, iNKT cell-surface receptors were down-regulated, which rendered iNKT cells invisible in flow cytometric detection (38, 39). In the current study, the numbers of hepatic iNKT cells decreased with time after hepatic I/R injury in mice with or without α-GalCer treatment. The downregulation of TCRβ in α-GalCer-treated mice continued until at least 48 h after reperfusion. Hepatic iNKT cells in α-GalCer-treated mice were re-expanded at 72 h and 96 h post-reperfusion, which is consistent with the results showing that iNKT cells re-appeared at 72 h and 96 h after only administration of α-GalCer to the liver (38).

As shown in **Figure 2A**, the expression of genes related to pro-inflammatory and reparative macrophages in livers of α-GalCer-treated mice was up-regulated as early as 6 h post-reperfusion. The results indicate that activated iNKT cells may induce the accumulation of pro-inflammatory and reparative macrophages in the liver during the early phase of hepatic I/R injury. Therefore, activated iNKT cells accelerate a switch in the macrophage phenotype. This hypothesis was supported by results from flow cytometry analysis which showed that α-GalCer treatment induced early switching from a pro-inflammatory to a reparative macrophage phenotype.

In this study, we demonstrated that activation of iNKT cells induced accelerated polarization of macrophages to facilitate liver repair after hepatic I/R injury. We also showed that the absence of iNKT cells delayed the transition of macrophage phenotypes, which was associated with delayed liver repair. Activated iNKT cells produced IL-4 at 6 h post-reperfusion. Anti-IL-4 antibody treatment delayed the switch of Ly6C^high^ macrophages to Ly6C^low^ macrophages during hepatic I/R injury, and suppressed the induction of hepatocyte proliferation to and healing of the injured site. Further studies are required to analyze whether IL-4 produced from iNKT cells mediates macrophage polarization to promote liver repair after hepatic I/R injury.

Consistent with this, IL-4 inhibition delays the differentiation of monocyte-derived macrophages into an anti-inflammatory and tissue reparative phenotype, which affects the repair process induced by sterile thermal injury in the liver (32). During bacterial infection, basophil-derived IL-4 promotes the switch of Ly6C^high^ monocyte-derived macrophages to anti-inflammatory monocyte-derived macrophages, thereby resolving inflammation and restoring tissue homeostasis in the liver (40). A recent report suggested that accumulation of iNKT cells in damaged liver tissues induced by focal sterile thermal injury is involved in tissue repair (19). The iNKT cell responses in our study correlated with a switch from inflammatory monocytes to reparative monocytes around the lesion, which was dependent on the IL-4 produced by iNKT cells.

In addition to IL-4, IL-13 is involved in reparative macrophage polarization to resolve inflammation and for wound healing of damaged tissues in the liver (41). The current results demonstrate that α-GalCer treatment increased hepatic levels of *Il13* and *Il4*, suggesting that IL-13 contributes to liver repair after hepatic I/R injury. However, activated iNKT cells produced lesser IL-13 than IL-4 and IFN-γ, suggesting that sources other than iNKT cells are involved in IL-13 production during hepatic I/R injury in α-GalCer-treated mice. We have previously reported that IL-13 derived from dendritic cells facilitates liver restoration following hepatic I/R injury (42). Further studies are necessary to elucidate the role of IL-13 in iNKT cell-induced liver repair after hepatic I/R injury.

IFN-γ also contributed to macrophage transition to a pro-inflammatory phenotype. Our data showed that iNKT cells activated by α-GalCer produced IFN-γ during the early phase of hepatic I/R injury. The current study also demonstrated that IFN-γ blockade reduced the accumulation of Ly6C^high^ macrophages, suggesting that iNKT cell-derived IFN-γ stimulated the accumulation of Ly6C^high^ macrophages. *In vitro* studies indicated that IFN-γ blockade decreased the number of pro-inflammatory macrophages in co-culture with activated iNKT cells. These results suggest that IFN-γ promotes the transition of macrophages into a pro-inflammatory phenotype.

On the other hand, IFN-γ blockade attenuated liver injury and pro-inflammatory mediators, which were associated with stimulation of liver repair and hepatocyte proliferation, suggesting that IFN-γ derived from activated iNKT cells increases liver inflammation and decreases liver repair after hepatic I/R injury. In addition, Ly6C^high^ macrophages may be one of the initiators of the acute phase of hepatic I/R injury, which contributes to delayed liver repair (3). Consistent with this, inhibition of IFN-γ protects against hepatic I/R injury in mice (14). Increased levels of IFN-γ stimulate hepatic inflammation and exacerbate liver damage, leading to increase in hepatic fibrosis (43, 44).

Our data revealed that α-GalCer treatment resulted in the production of pro-inflammatory and anti-inflammatory cytokines, IFN-γ and IL-4, simultaneously from activated iNKT cells. Both IFN-γ and IL-4 play a role in polarizing macrophages, and stimulation of macrophages with IFN-γ or IL-4 initiates the transition of pro-inflammatory or reparative macrophages, respectively. Indeed, in this study, IFN-γ polarized macrophages into a pro-inflammatory phenotype, while IL-4 polarized them into a reparative phenotype, indicating variation in the effects of cytokines on macrophage polarization. Despite the ability of IFN-γ to enhance hepatic I/R injury, our data showed that α-GalCer-treated mice exhibited enhanced resolution of inflammation and liver repair after hepatic I/R injury. These results suggest that IFN-γ contributes to liver repair to a lesser extent than IL-4 after hepatic I/R. Although IFN-γ was found to be involved in liver injury and inflammation in this study, the role of IFN-γ in liver inflammation and repair in this model warrants further study. Additionally, IFN-γ inhibition reduced the accumulation of Ly6C^high^ macrophages; however, it did not suppress the accumulation of Ly6C^low^ macrophages. Hepatic levels of *Il4* remained high in anti-IFN-γ antibody-treated mice as well as in control IgG-treated mice. These results suggest that IFN-γ does not affect IL-4-mediated macrophage transition to a reparative phenotype.

The current study demonstrated that activation of iNKT cells by α-GalCer facilitated hepatocyte proliferation in the non-injured regions of the liver after hepatic I/R injury. We also showed that α-GalCer alone did not affect hepatocyte proliferation in the liver. However, others have suggested that activation of iNKT cells by α-GalCer suppresses liver regeneration after partial hepatectomy in mice, which is mediated by IFN-γ and IL-4 (20). These discrepant results may be due to the difference in experimental models. In hepatic I/R injury, severe liver injury is induced, followed by the recruitment of inflammatory cells, including neutrophils, monocytes, and macrophages. In contrast, in partial hepatectomy, minimal liver injury is induced, associated with the proliferation of resident liver macrophages and lesser with the accumulation of inflammatory cells.

Stimulation of iNKT cells with α-GalCer influences liver inflammation and repair through interactions with macrophages. Consistent with this, activated iNKT cells promote the resolution of inflammation and tissue repair after myocardial infarction in mice by affecting macrophage infiltration (17). *In vivo* stimulation of adipose iNKT cells with α-GalCer leads to the production of IL-10 and IL-4, which can induce a reparative macrophage phenotype in macrophages (45). The present results of *in vitro* studies revealed that iNKT cells activated by α-GalCer differentiate macrophages into a pro-inflammatory phenotype as well as a reparative macrophage phenotype through the production of IL-4 and IFN-γ. These results suggest that the interaction between iNKT cells and macrophages is important for modulating macrophage polarization. *In vitro* studies showed that there was no change in the expression of pro-inflammatory and reparative macrophage-related genes (except for *Nos2*) in macrophages co-cultured with iNKT cells in the absence of α-GalCer, indicating that direct contact alone of macrophages with iNKT cells did not up-regulate the expression of these genes in macrophages. It is unclear why *Nos2* mRNA levels were greater in naïve macrophages co-cultured with sorted iNKT cells (TCRβ^+^α-GalCer tetramers^+^). α-GalCer/CD1d tetramers which were used for sorting iNKT cells may partly activate iNKT cells, or the potential presence of endogenous ligands to NKT cells in naïve macrophages may affect iNKT cells in culture (24).

In addition, *in vitro* studies indicated that neutralizing antibodies against IL-4 up-regulated pro-inflammatory macrophage-related genes; however, several genes including *Il6* and *Nos2* were down-regulated. These results indicate that blockade of IL-4 alone fails to polarize macrophages into a pro-inflammatory phenotype or that cytokines other than IL-4 released from iNKT cells may down-regulate the expression of these genes in the co-culture system. Our data showed that IL-4 antibodies down-regulated the expression of *Ifng* genes in iNKT cells. Although the underlying mechanism is unclear, down-regulation of *Ifng* in iNKT cells may down-regulate the expression of genes including *Il6* and *Nos2*. A similar result was observed for treatment with neutralizing antibodies against IFN-γ, showing that blockade of IFN-γ did not up-regulate all reparative macrophage-related genes. In addition, the current study showed that IFN-γ antibodies up-regulated the levels of *Il4* mRNA in iNKT cells. These results suggest that, in addition to IL-4, other factors may be required for the polarization of macrophages into a reparative phenotype in the co-culture system.

In the liver, CD1d is expressed on sinusoidal endothelial cells, Kupffer cells, hepatocytes, and other cells, including hepatic stellate cells and circulating DCs (11, 12, 19). In the current study, immunofluorescence analyses revealed that CD1d expression, at least in part, co-localized with macrophages in the livers treated with α-GalCer, which is consistent with the results of flow cytometric analyses. Because iNKT cells do not infiltrate into the injured regions during the early phase of acute sterile liver injury (19), the cells might directly interact with macrophages, but not with hepatocytes, during the early phase of hepatic I/R injury. Further, CD1d expression in macrophages was up-regulated in the presence of activated iNKT cells *in vitro*, suggesting that macrophages expressing CD1d present α-GalCer to activate iNKT cells. Overall, iNKT cells contribute to polarizing macrophages that accumulate in the injured regions through the interaction with CD1d-expressing macrophages.

Interestingly, iNKT cell-deficient mice *Cd1d^-/-^* mice showed delayed conversion of the macrophages, decreased cellular proliferation, and decreased liver repair. These results suggest that iNKT cells play a role in liver repair after hepatic I/R injury, and that macrophage transition is independent of the presence of iNKT cells. Enhanced *Ifng* expressions from cells other than iNKT cells increased the number of pro-inflammatory macrophages and decreased the number of reparative macrophages, which resulted in delayed liver repair. *Cd1d^-/-^* mice lack CD1d-restricted NKT cells, which include type I NKT or iNKT cells and type II NKT or diverse NKT cells (46). Type I NKT cells, which recognize α-GalCer, express semi-invariant TCRs, whereas type II NKT cells express more diverse TCRs. Our results indicate that type I NKT cells are involved in liver repair after hepatic I/R injury. However, the results from *Cd1d^-/-^* mice do not exclude the involvement of type II NKT cells. Previously, activation of type II NKT cells by sulfatide was shown to attenuate hepatic I/R injury by reducing IFN-γ production from type I NKT cells in mice (37). Therefore, further studies are required to evaluate the involvement of type II NKT cells in liver repair after hepatic I/R injury.

In conclusion, our data demonstrate that iNKT cells activated by α-GalCer produce IL-4 to enhance macrophage phenotype switching, which results in the promotion of liver repair after hepatic I/R injury. In addition, IFN-γ derived from activated iNKT cells accelerated polarization of pro-inflammatory macrophages followed by reparative macrophage polarization, but displayed a limited role in inducing liver inflammation during hepatic I/R injury in mice treated with α-GalCer. These results suggest that the interaction of iNKT cells with macrophages determines the extent and duration of hepatic I/R injury, followed by that of liver repair. Therefore, therapies designed to activate iNKT cells may represent a therapeutic tool for liver repair after hepatic I/R injury to improve the prognosis of liver surgery.

## Data Availability Statement

The original contributions presented in the study are included in the article/**Supplementary Material**. Further inquiries can be directed to the corresponding author.

## Ethics Statement

The animal study was reviewed and approved by Kitasato University School of Medicine.

## Author Contributions

TG: conceived, designed, and performed the experiments, and wrote the manuscript. YI: conceived, designed, and performed the experiments, and wrote the manuscript. MS: performed the experiments, and performed data analysis and interpretation. SN: performed the experiments and provided technical support. NN: performed the experiments and provided technical support. KH: performed the experiments and provided technical support. TN: performed data analysis and interpretation. KE: performed data analysis, and interpretation, and provided technical support. KI: performed data interpretation, provided technical support and revised the manuscript critically for intellectual content. NH: verified the results of the experiments and revised the manuscript critically for intellectual content. HA: conceived the project and supervised the study. All authors contributed to the article and approved the submitted version.

## Funding

This research was supported by research grants from the Ministry of Education, Culture, Sports, Science, and Technology of Japan (No. 19K09156 to YI, No. 20K17630 to NN), an Integrative Research Program of the Graduate School of Medical Science, Kitasato University, and Parents’ Association Grant of Kitasato University School of Medicine.

## Conflict of Interest

The authors declare that the research was conducted in the absence of any commercial or financial relationships that could be construed as a potential conflict of interest.

## Publisher’s Note

All claims expressed in this article are solely those of the authors and do not necessarily represent those of their affiliated organizations, or those of the publisher, the editors and the reviewers. Any product that may be evaluated in this article, or claim that may be made by its manufacturer, is not guaranteed or endorsed by the publisher.
