# Supplementary material for: Activation of iNKT Cells Facilitates Liver Repair After Hepatic Ischemia Reperfusion Injury Through Acceleration of Macrophage Polarization
Source: Front Immunol. 2021 Oct 6;12:754106. doi: 10.3389/fimmu.2021.754106 (PMC8526965; doi:10.3389/fimmu.2021.754106)
Supplement: Supplementary file 1 [file DataSheet_1.pdf]

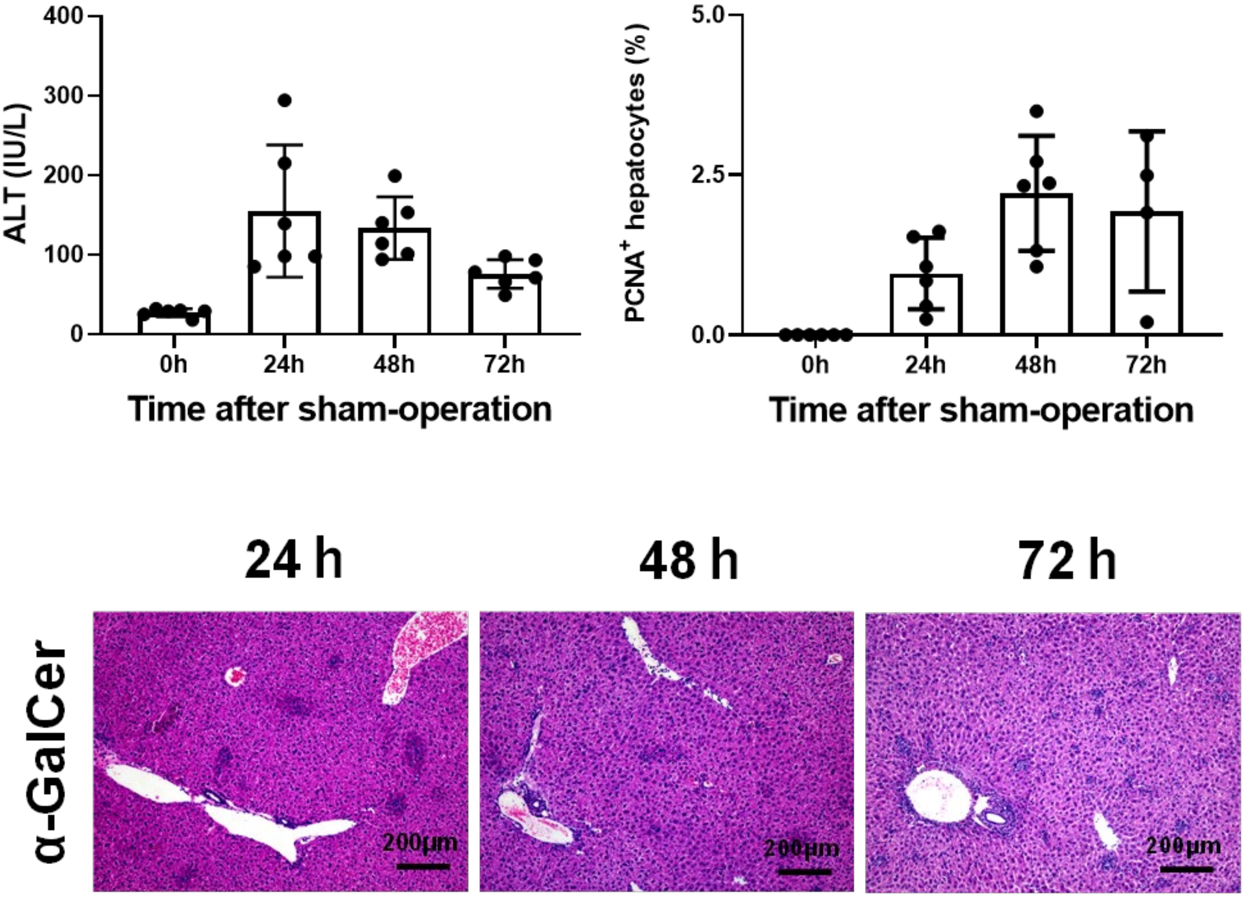

Supplementary Figure S1.

Liver injury and hepatocyte proliferation in sham-operated mice treated with α-GalCer. ALT levels, representative photomicrographs of H&E-stained liver sections, and $PCNA^{+}$-hepatocytes (%) after administration of α-GalCer to sham-operated mice. Data are expressed as mean ± SD (four–six biological replicates per group). Scale bars, 200 μm.

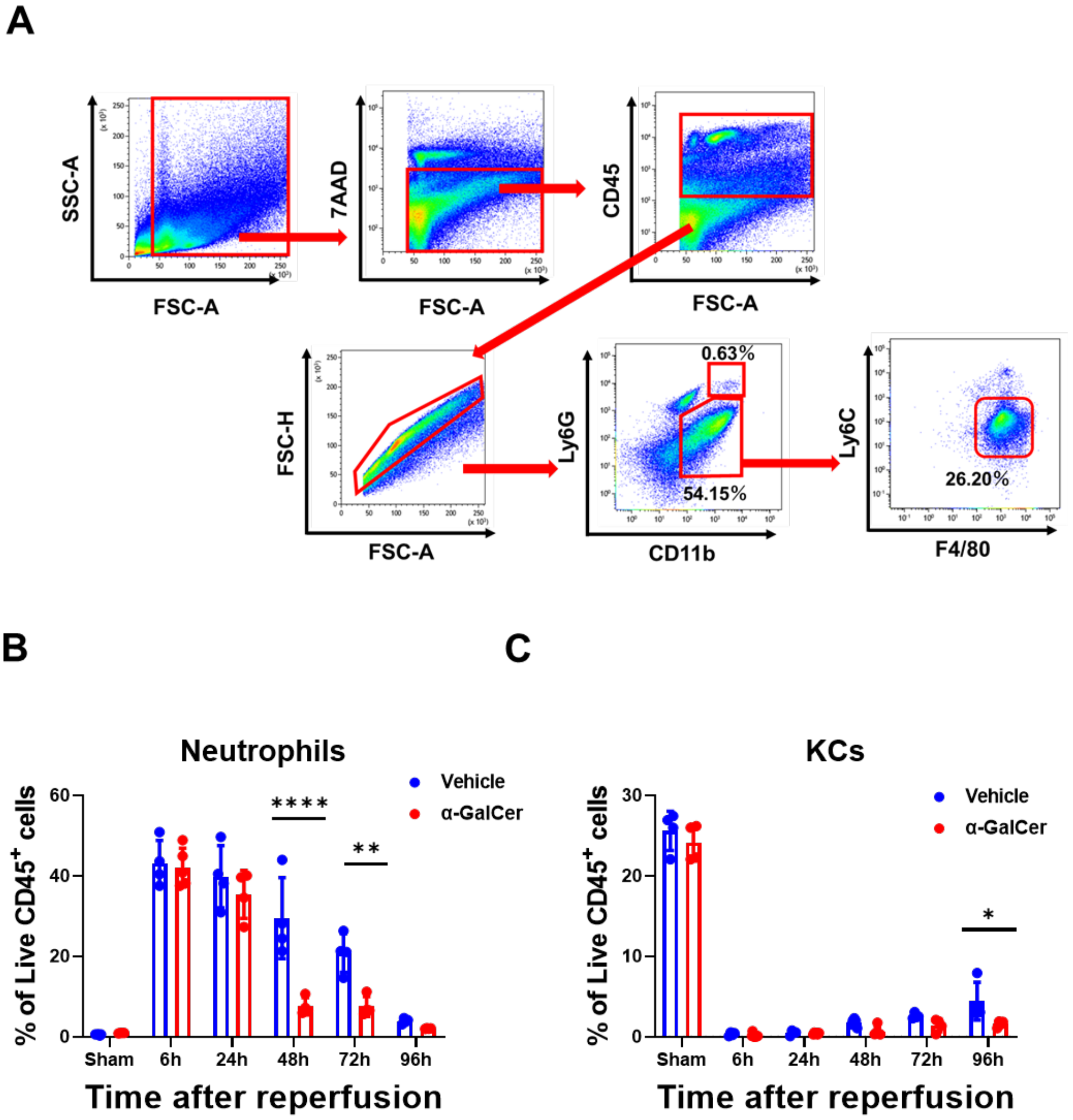

Supplementary Figure S2. Infiltration of immune cells after hepatic I/R injury.
(A) Flow cytometry gating strategy used to identify neutrophils and Kupffer cells (KCs) in livers from sham-operated mice. (B,C) Changes in the percentage of neutrophils (B) and KCs (C) in livers from mice treated with α-GalCer or vehicle after hepatic I/R injury. Neutrophils were defined as CD45+/Ly6G+/CD11b+ cells; KCs, CD45+Ly6G^{low}/Ly6C-/CD11b^{low}/F4/80^{high} -cells. Data are expressed as the mean ± SD (four biological replicates per group). * P <0.05, ** P <0.01, **** P <0.0001.

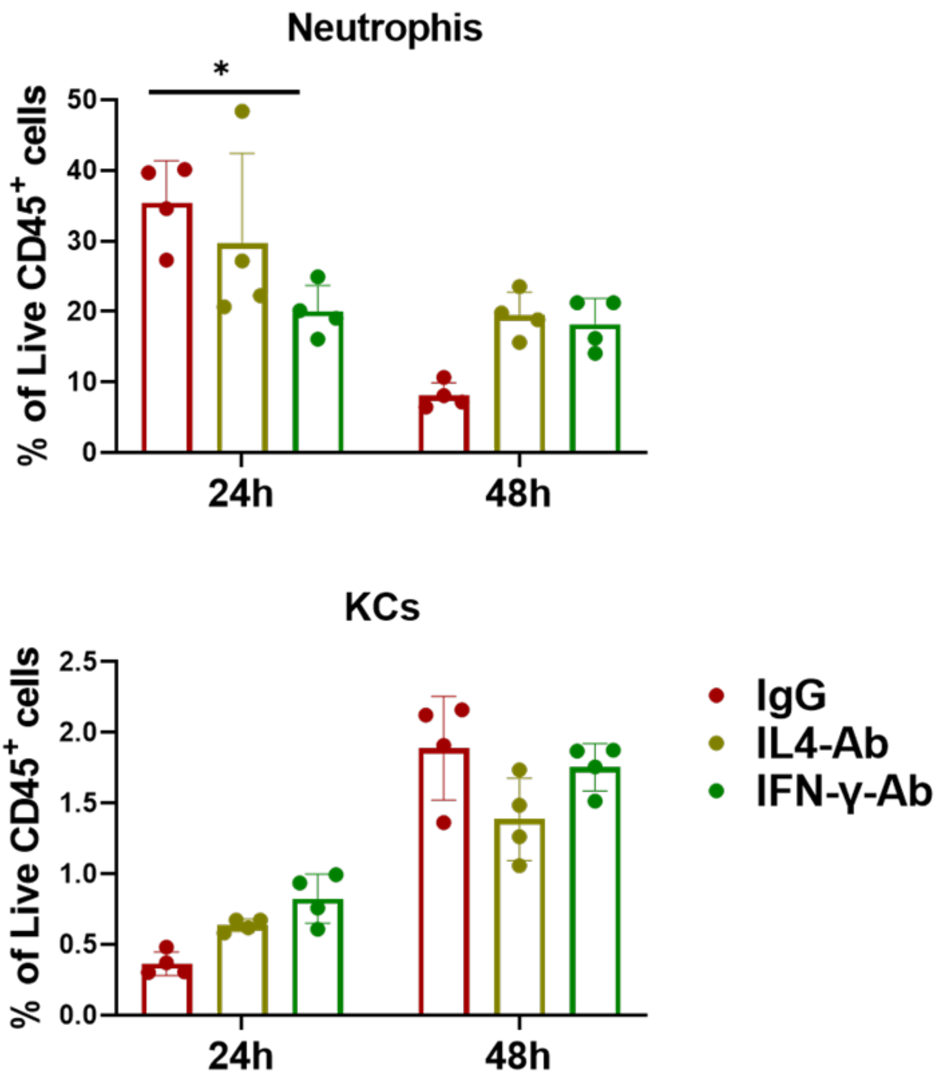

Supplementary Figure S3.

Effect of IL-4 or IFN-γ blockade on the percentage of neutrophils and Kupffer cells after hepatic I/R injury. Anti-IL-4 or IFN-γ neutralizing antibodies were administered to mice 1 h prior to the induction of ischemia, and α-GalCer was given at the induction of ischemia. The percentage of neutrophils and Kupffer cells in livers from mice treated with anti-IL-4 antibodies and IgG. Neutrophils and Kupffer cells are defined as CD45+/Ly6G+/CD11b+ cells and CD45+Ly6G^{low}/Ly6C-/CD11b^{low}/F4/80^{high} -cells, respectively. Data are expressed as the mean ± SD (four biological replicates per group). * P <0.05.

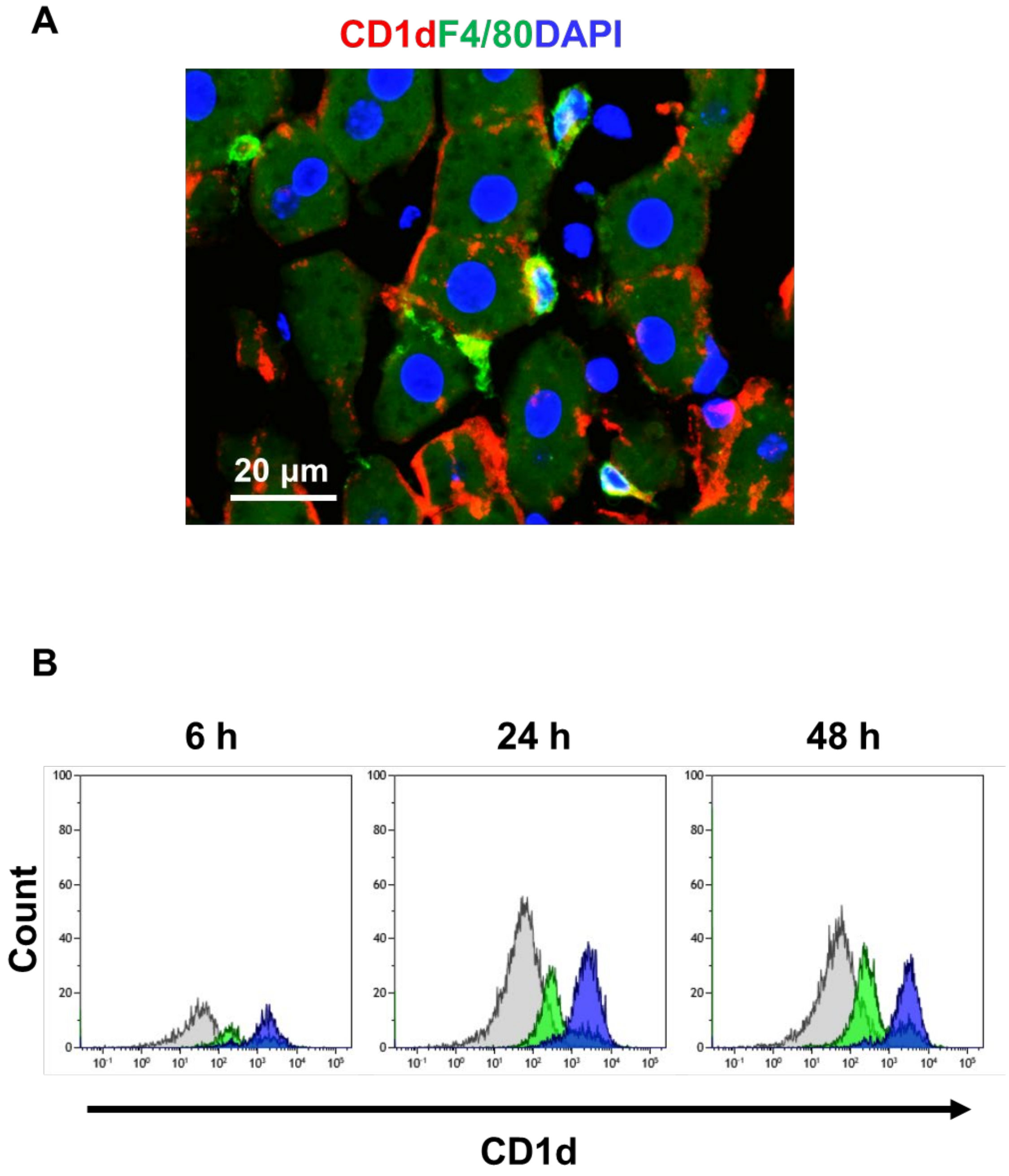

Supplementary Figure S4.

CD1d expression in the livers after hepatic I/R injury treated with α-GalCer.

(A) Representative photo showing double staining of CD1d (red) and F4/80 (green) in livers treated with α-GalCer at 6 h post-reperfusion. Yellow indicates double-positive cells. Nuclei are stained by DAPI (blue). Scale bar, 20 µm. (B) The expression of CD1d in Ly6C^{high} macrophages (Ly6G^{-}/Ly6C^{high}/CD11b^{high}/F4/80^{high} cells) and Ly6C^{low}macrophages (Ly6G^{-}/Ly6C^{low}/CD11b^{high}/F4/80^{high} cells) at 6, 24, and 48 h post-reperfusion (light gray, isotype control; light green, Ly6C^{low}macrophages and Ly6C^{high} macrophages, light blue).

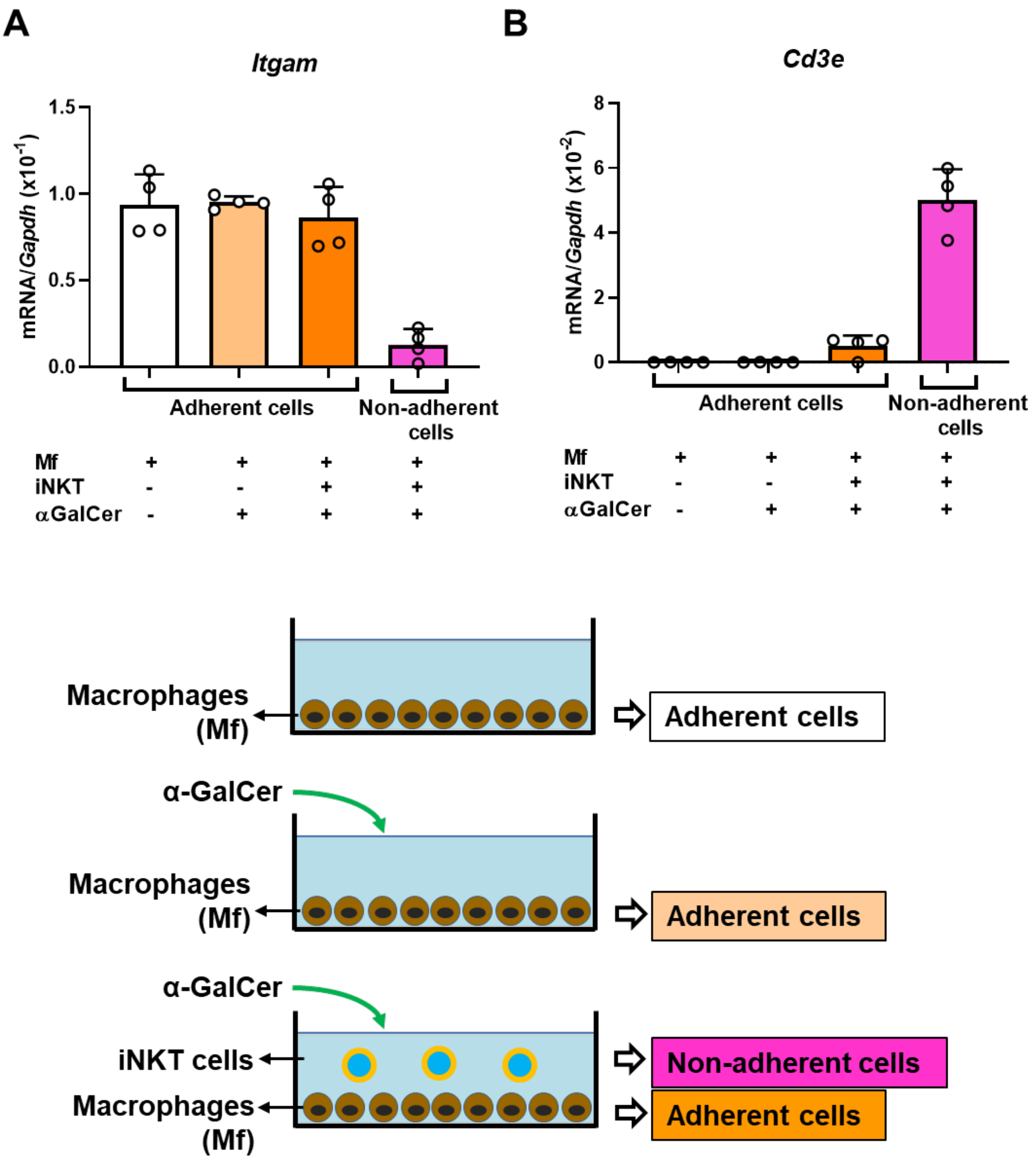

Supplementary Figure S5.

Characterization of adherent and non-adherent cells in co-culture of macrophages and activated iNKT cells.

(A,B) Expression of mRNA for *Itgam* (A) and *Cd3e* (B) in adherent and non-adherent cells in co-culture of macrophages and iNKT cells. Cultured bone marrow-derived macrophages and isolated iNKT cells were stimulated with α-GalCer for 72 h. Then, mRNA levels of *Itgam* and *Cd3e* were determined from adherent and non-adherent cells to the wells. Data are expressed as the mean ± SD (four mice per group, representative data from two independent experiments).

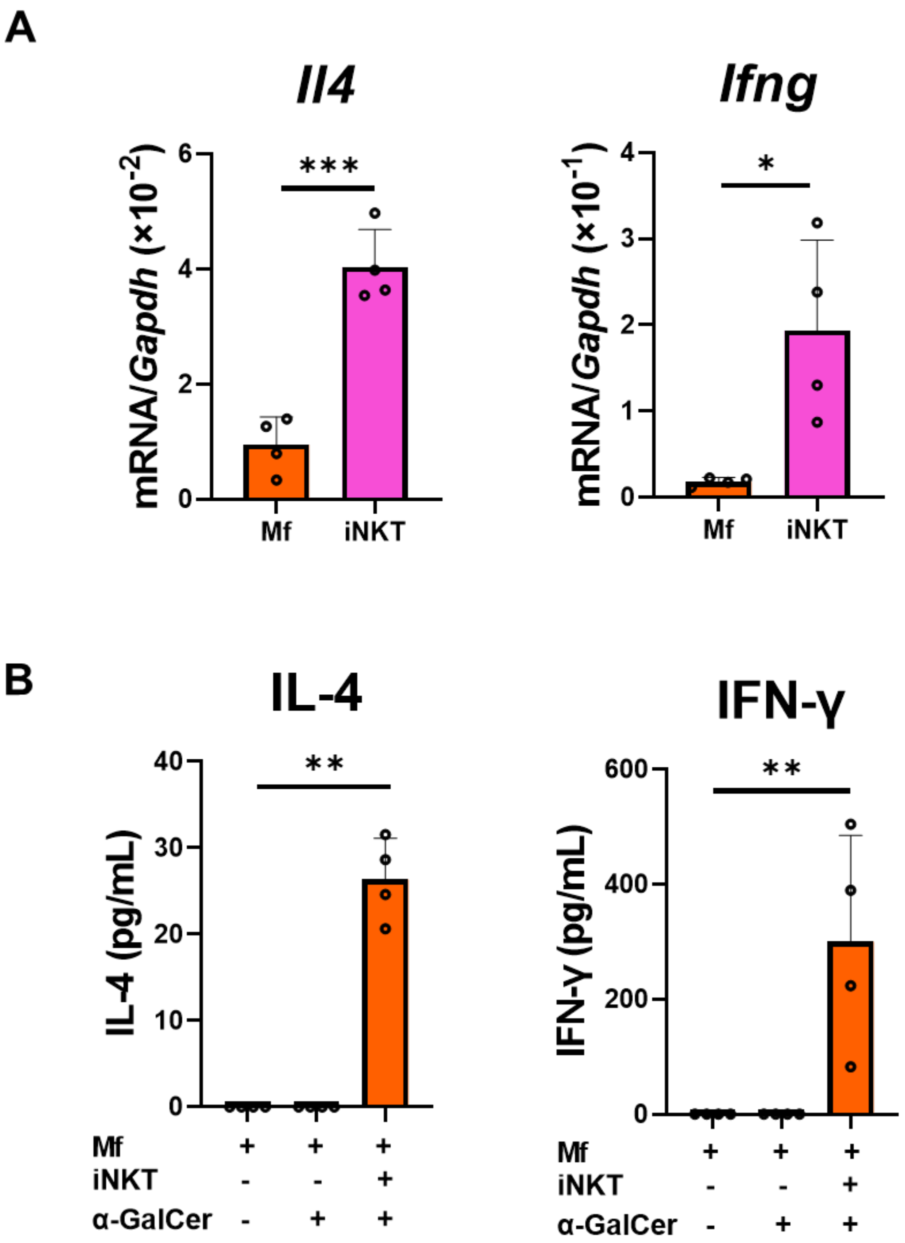

Supplementary Figure S6.

Production of IL-4 and IFN-γ from iNKT cells in vitro.

(A) The levels of mRNA for *Il4* and *Ifng* from cultured activated iNKT cells and macrophages. Mf, macrophages. Data are expressed as the mean ± SD (four mice per group, representative data from two independent experiments). * P <0.05, *** P <0.001.

(B) The levels of IL-4 and IFN-γ in supernatants from activated iNKT cells and macrophages. Mf, macrophages. Data are expressed as the mean ± SD (four mice per group, representative data from two independent experiments). ** P <0.01.

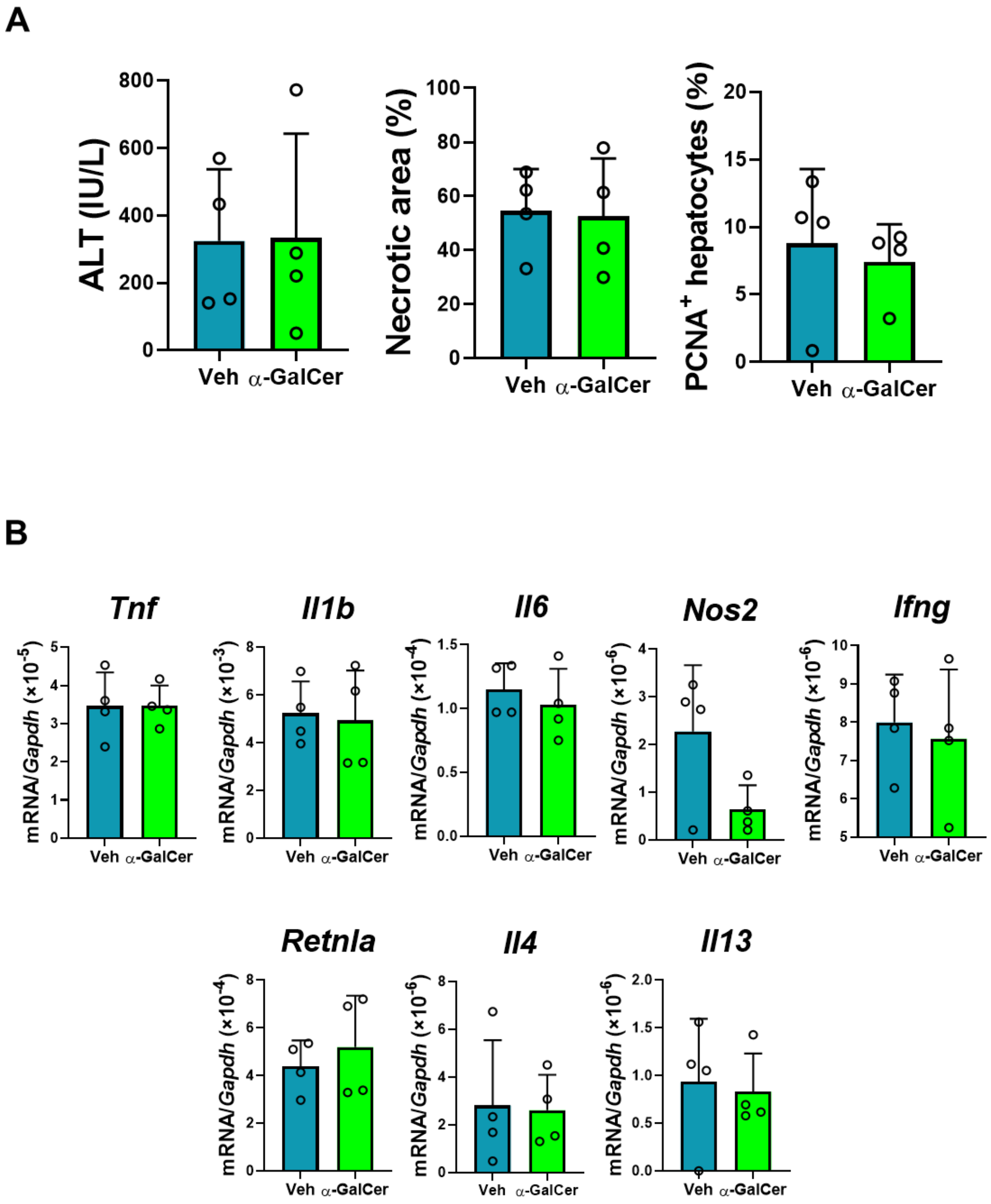

Supplementary Figure S7.

Liver repair after hepatic I/R injury in *Cd1d*$^{-/-}$ mice treated with α-GalCer or vehicle.

(A) ALT levels, hepatic necrosis, and PCNA^{+}-hepatocytes (%), and (B) expression of genes related to pro-inflammatory (*Tnf, Il1b, Il6, Nos2, and Ifng*) and reparative macrophages phenotypes (*Mrc1, Retnla, Il4, and Il13*) at 72 h post-reperfusion. Data are expressed as the mean ± SD (four biological replicates per group). Veh, vehicle.
